# Hydrogen Therapy Reverses Cancer‐Associated Fibroblasts Phenotypes and Remodels Stromal Microenvironment to Stimulate Systematic Anti‐Tumor Immunity

**DOI:** 10.1002/advs.202401269

**Published:** 2024-05-17

**Authors:** Xiaoyan Meng, Zhonglong Liu, Liang Deng, Yangzi Yang, Yingchun Zhu, Xiaoying Sun, Yongqiang Hao, Yue He, Jingke Fu

**Affiliations:** ^1^ Department of Oral Maxillofacial & Head and Neck Oncology Shanghai Ninth People's Hospital Shanghai Jiao Tong University School of Medicine Shanghai 200011 P. R. China; ^2^ College of Stomatology National Center for Stomatology National Clinical Research Center for Oral Diseases Shanghai Key Laboratory of Stomatology Shanghai Jiao Tong University Shanghai 200011 P. R. China; ^3^ Shanghai Key Laboratory of Orthopaedic Implant Department of Orthopaedic Surgery Shanghai Ninth People's Hospital Shanghai Jiao Tong University School of Medicine Shanghai 200011 P. R. China; ^4^ Clinical and Translational Research Center for 3D Printing Technology Shanghai Engineering Research Center of Innovative Orthopaedic Instruments and Personalized Medicine Shanghai 200011 P. R. China; ^5^ Department of Orthopedic Surgery Spine Center Changzheng Hospital Navy Medical University No. 415 Fengyang Road Shanghai 200003 P. R. China; ^6^ Key Laboratory of Inorganic Coating Materials Shanghai Institute of Ceramics Chinese Academy of Sciences Shanghai 200050 P. R. China; ^7^ College of Sciences Shanghai University Shanghai 200444 P. R. China

**Keywords:** cancer‐associated fibroblasts, hydrogen therapy, immunotherapy, tumor microenvironment

## Abstract

Tumor microenvironment (TME) plays an important role in the tumor progression. Among TME components, cancer‐associated fibroblasts (CAFs) show multiple tumor‐promoting effects and can induce tumor immune evasion and drug‐resistance. Regulating CAFs can be a potential strategy to augment systemic anti‐tumor immunity. Here, the study observes that hydrogen treatment can alleviate intracellular reactive oxygen species of CAFs and reshape CAFs’ tumor‐promoting and immune‐suppressive phenotypes. Accordingly, a controllable and TME‐responsive hydrogen therapy based on a CaCO_3_ nanoparticles‐coated magnesium system (Mg‐CaCO_3_) is developed. The hydrogen therapy by Mg‐CaCO_3_ can not only directly kill tumor cells, but also inhibit pro‐tumor and immune suppressive factors in CAFs, and thus augment immune activities of CD4^+^ T cells. As implanted in situ, Mg‐CaCO_3_ can significantly suppress tumor growth, turn the “cold” primary tumor into “hot”, and stimulate systematic anti‐tumor immunity, which is confirmed by the bilateral tumor transplantation models of “cold tumor” (4T1 cells) and “hot tumor” (MC38 cells). This hydrogen therapy system reverses immune suppressive phenotypes of CAFs, thus providing a systematic anti‐tumor immune stimulating strategy by remodeling tumor stromal microenvironment.

## Graphical abstract

Meng et al. observe that hydrogen treatment can alleviate intracellular reactive oxygen species of cancer‐associated fibroblasts (CAFs) and reshape CAFs’ tumor‐promoting and immune‐suppressive phenotypes. They develop a controllable and tumor microenvironment‐responsive hydrogen therapy based on a CaCO_3_ nanoparticles‐coated magnesium system (Mg‐CaCO_3_), which targets both tumor cells and CAFs, and stimulates systematic anti‐tumor immunity by remodeling tumor stromal microenvironment.

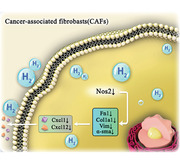

## 1 Introduction

Gas therapy, an emerging and promising treatment method, has attracted increasing attention for anti‐tumor treatments.^[^ ^1^ ^]^ As endogenous gasotransmitters, gas molecules such as nitric oxide (NO), carbon monoxide (CO), hydrogen sulfide (H_2_S), and hydrogen (H_2_) could regulate biological process of tumor cells, including vasodilatation, neurotransmission, proliferation, and metastasis.^[^ ^2^ ^]^ The commonly accepted mechanism of their anti‐tumor effects is that these gasotransmitters bind to haem iron centers in various proteins, especially hemoglobin in mitochondria, inducing bioenergetics disorder in tumor cells.^[^ ^3^ ^]^ Among numerous therapeutic gases, H_2_ has no blood poisoning risk even at high concentrations, and shows a significant safety benefit.^[^ ^4^, ^5^ ^]^ In recent years, hydrogen therapy was widely utilized in the development of novel nanomedicine anti‐tumor strategies.^[^ ^6^, ^7^, ^8^, ^9^, ^10^, ^11^, ^12^ ^]^ However, these therapeutic strategies mainly focused on cancer cells themselves while comprehensive therapeutic effects of hydrogen gas on the TME left to be elucidated.

Among TME components, CAFs generate in multiple tumor‐promoting effects and could induce therapy non‐response and drug‐resistance.^[^ ^13^, ^14^ ^]^ Fibroblasts are recruited and activated by tumor cells. In turn, they produce various extracellular matrix (ECM) proteins to build up the physical barrier that impedes drug delivery and T‐cell infiltration.^[^ ^15^, ^16^ ^]^ In addition, CAFs also secrete large quantities of growth factors, cytokines, and chemokines, such as transforming growth factor‐β (TGF‐β), interleukin‐6 (IL‐6), and CC‐chemokine ligand 2 (CCL2), to reduce T‐cell response and recruit immunesuppressive cells (e.g., Tregs, myeloid‐derived suppressor cells, etc.), assisting in tumor immune evasion.^[^ ^16^, ^17^, ^18^ ^]^ Therefore, CAFs might be a promising target for anti‐tumor therapies, and regulating CAFs could be a potential strategy to augment systemic anti‐tumor immunity. Actually, multiple nanoparticle therpeutics regulating CAFs have been developed. Nicolás‐Boluda et al. removed CAFs in TME through the photothermal effect of gold nanoparticles, blocked tumor stromal remodeling process, reduced tumor stiffness, and thus inhibited tumor growth.^[^ ^19^ ^]^ Li et al. regulated CAFs to reduce tumor ECM and enhance immune infiltration with tumor cell‐derived microparticles.^[^ ^20^ ^]^ Zhou et al. developed peptide nano‐blanket to inhibit fibroblasts activation and reverse metastasis‐supportive stromal tissue.^[^ ^21^ ^]^ Recently, Guo et al. addressed metabolic reprogramming and epigenetic regulation of CAFs by nano‐platform and improved tumor responses to chemotherapy.^[^ ^22^ ^]^ Wang et al. constructed a sequential nanocomposite hydrogel to reshape CAFs behavior and enhance T‐cell infiltration in osteosarcoma.^[^ ^23^ ^]^ All these studies highlight the promising application perspectives of CAFs‐targeting anti‐tumor therapeutics.

In this study, we observed that hydrogen treatment could alleviate intracellular reactive oxygen species (ROS) of CAFs, disturb the transcriptional program through down‐regulating nitric oxide synthase (*Nos2*) expression level, and reverse CAFs’ tumor‐promoting and immune suppressive phenotypes. According to this result, we developed a controllable and TME‐responsive hydrogen therapy based on CaCO_3_ nanoparticles‐coated magnesium (Mg) rods (Mg‐CaCO_3_). We found that the Mg‐CaCO_3_ exhibited rapid and stable hydrogen release capability in the weak acid TME, reduced pro‐tumor and immune suppressive factor generation of CAFs including Cxcl1 and Cxcl12, and augmented anti‐tumor immune activities of CD4^+^ T cells. Moreover, in the bilateral tumor transplantation model of 4T1 and MC38 cells, we observed higher systematic immune stimulating factors in mice serum and higher immune infiltration in the distant tumors, indicating the superior systematic immune activation effects of hydrogen therapy by Mg‐CaCO_3_.

## 2 Results

### 2.1 CAFs Shape Cold Tumors while Hydrogen Treatment Remodels CAFs’ Phenotypes

**Figure 1:**
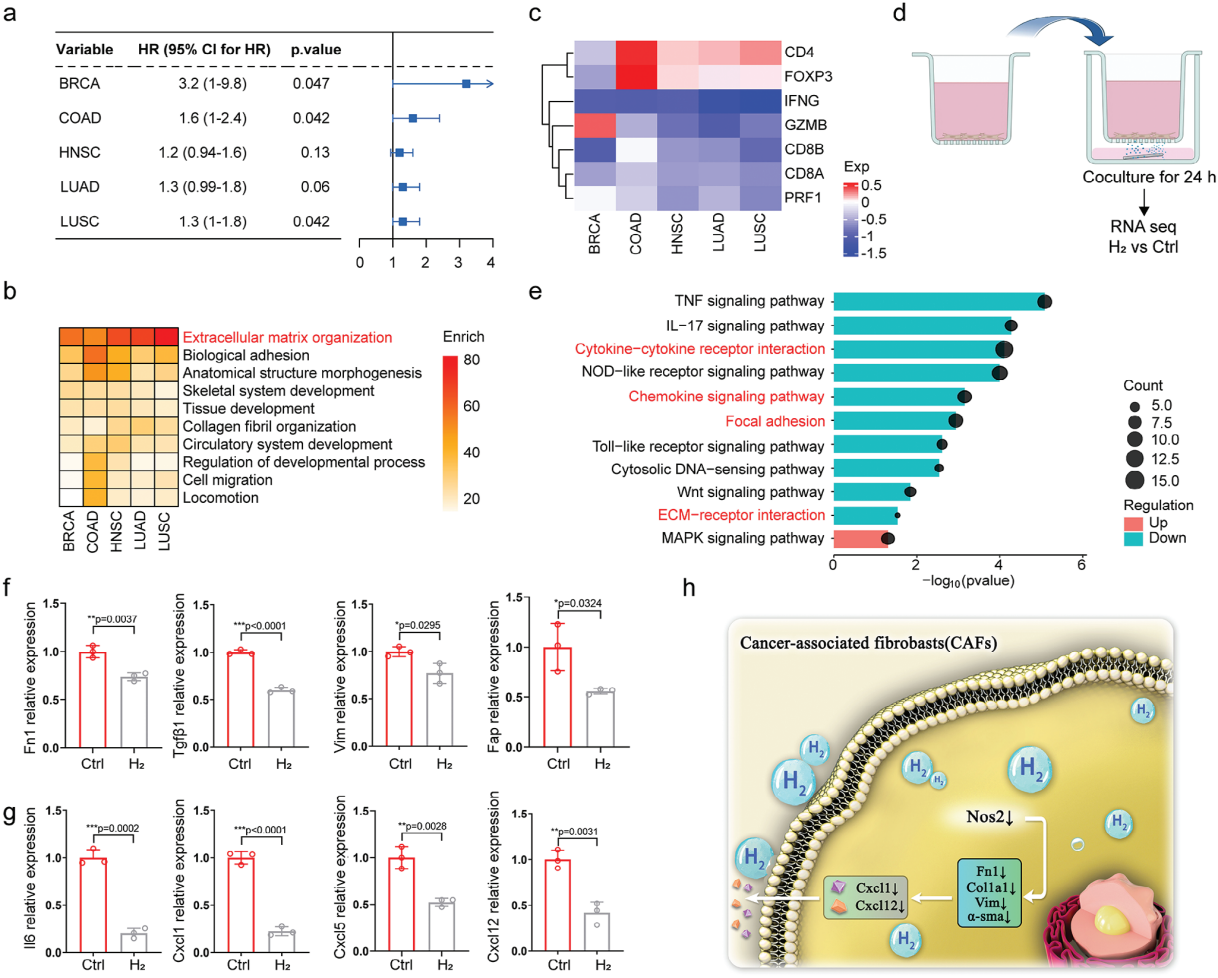
Profiling of CAFs in pan‐cancer and CAFs’ phenotypes reverse by hydrogen treatment. a) The HR for overall survival (OS) between the high‐ and low‐ CAF signature expression groups in different TCGA datasets. b) The heatmap shows enriched pathways of up‐regulated genes in CAF‐high groups in different TCGA datasets. c) The heatmap shows key immune genes expression fold change between CAF‐high and CAF‐low groups in different TCGA datasets. d) Schematic illustration of the in vitro hydrogen treatment model using transwell (created with bioRender.com). e) Bar plots show enriched pathways of DEGs in H_2_ group compared to Ctrl group. f,g) MRNA levels of CAF markers (f) and immune‐modulatory factors (g) analyzed by qPCR in CAFs with (H_2_) or without (Ctrl) hydrogen treatment (*n* = 3). h) Schematic illustration of CAFs’ phenotypes reversing mechanism of hydrogen treatment. Statistical significance was calculated via two‐tailed Students’ *t*‐test. ^*^ *p* < 0.05, ^**^ *p* < 0.01, and ^***^ *p* < 0.001. The mean values and SD are presented.

Since CAFs play an important role in shaping the TME and promoting tumor progression, we performed a series of analyses regarding CAFs in TCGA datasets. Here, datasets of five major cancer types were included: breast cancer (BRCA), colon adenocarcinoma (COAD), head and neck squamous cell carcinoma (HNSC), lung adenocarcinoma (LUAD), and lung squamous cell carcinoma (LUSC). First, by survival analysis on CAFs signature, we observed hazard ratio (HR) > 1 in these cancer types (though *p* value in HNSC and in LUAD did not meet statistical significance, the results still showed a clear tendency), supporting that CAFs were pro‐tumor factors and were associated with poor outcomes (**Figure** **1**a). Then we divided patients into CAF‐high and ‐low groups according to the CAFs infiltration. By differentially expressed gene (DEG) analysis and functional enrichment, we found that high CAFs infiltration was most related to ECM organization (Figure 1b). According to current studies, CAFs are commonly considered to form multiple ECM components and to shape an immune‐excluded TME.^[^ ^24^ ^]^ In the meantime, CAFs also secret various cytokines and chemokines to generate immune suppressive cells like myeloid‐derived suppressor cells (MDSCs) and Tregs.^[^ ^13^ ^]^ In order to evaluate immune modulatory effects of CAFs, we then compared key immune genes expression levels between CAF‐high and ‐low groups in these five cancer types (Figure 1c). We observed consistent results in these datasets: in the CAF‐high group, *CD4* was up‐regulated with *FOXP3* expression, indicating CAFs were potentially associated with Treg infiltration. Additionally, *CD8* was down‐regulated with *IFNG*, *GZMB*, and *PRF1*, suggesting that CAFs could significantly inhibit CD8^+^ T cell infiltration and immune cytotoxicity. These results supported that CAFs play an important role in cancer progression through shaping an immune‐suppressive TME.

Based on the abovementioned analysis, we chose CAFs as our target to design anti‐tumor therapeutics. Here, we used a co‐culture model with the transwell device for hydrogen treatment (Figure 1d).^[^ ^25^ ^]^ Briefly, Mg rods (1 mm in diameter, 4 mm in length) were used as the hydrogen generator and laid into a 6‐well microplate filled with 1 mL medium in the well. CAFs were seeded in the transwell chamber and were fit into the 6‐well plates. In this way, hydrogen released by Mg rods could reach CAFs in the upper chamber while other factors like ions released into the medium will not affect them. By bulk RNA sequencing (RNA‐seq), we surprisingly observed that those CAFs treated with hydrogen significantly down‐regulated pro‐tumor and immune‐suppressive genes enriched in “ECM‐receptor interaction”, “Focal adhesion”, “Cytokine‐cytokine receptor interaction”, and “Chemokine signaling pathway” (Figure 1e). Key genes among these pathways included *Fn1*, *Mmp2*, *Il6*, *Cxcl1*, *Cxcl12*, etc., which were further validated by hydrogen treatment model and qPCR analysis (Figure 1f,g; Figure S1, Supporting Information).

We were also interested in the mechanism of hydrogen‐remodeling effects in CAFs, therefore, we evaluated DEGs between the control (Ctrl) and hydrogen (H_2_) group. And we noticed that *Nos2* was among the top down‐regulated genes after hydrogen treatment (Figure S2a, Supporting Information). Interestingly, after the same treatment with the model, *Nos2* mRNA expression level showed no significant change in tumor cells while showed significant downregulation in CAFs (downregulated to ca. 0.3) (Figure S2b, Supporting Information), indicating a CAF‐specific regulation effects of hydrogen gas. *Nos2* was a stress‐associated oncogene and was reported to induce multiple pro‐tumor factors in cancer.^[^ ^26^ ^]^ By using genetic engineering methods, we found that, after *Nos2* knockdown, expression levels of related biomarkers in CAFs would consistently decrease (Figure S3, Supporting Information). Moreover, in the rescue experiment, 1‐[N‐(2‐Aminoethyl)‐N‐(2‐ammonioethyl)amino]diazen‐1‐ium‐1,2‐diolate (DETA/NO) was added to the cell medium as a drug inducing *Nos2* expression after hydrogen treatment. And although consistent with current reports,^[^ ^26^ ^]^ *Fn1*, *Col1a1*, and *Vim* showed an inhibition trend in a high concentration, most pro‐tumor genes represented a dose‐dependent increase pattern (Figure S4, Supporting Information). These results collectively supported that CAFs play an important role in TME, shaping “cold” tumors and leading to poor clinical outcomes. And we found a potential therapeutic targeting CAFs: hydrogen treatment could reduce *Nos2* expression level on CAFs, further abating downstream pro‐tumor biomarkers (Figure 1h).

### 2.2 Preparation and Characterization of Mg‐CaCO_3_ Rods

**Figure 2:**
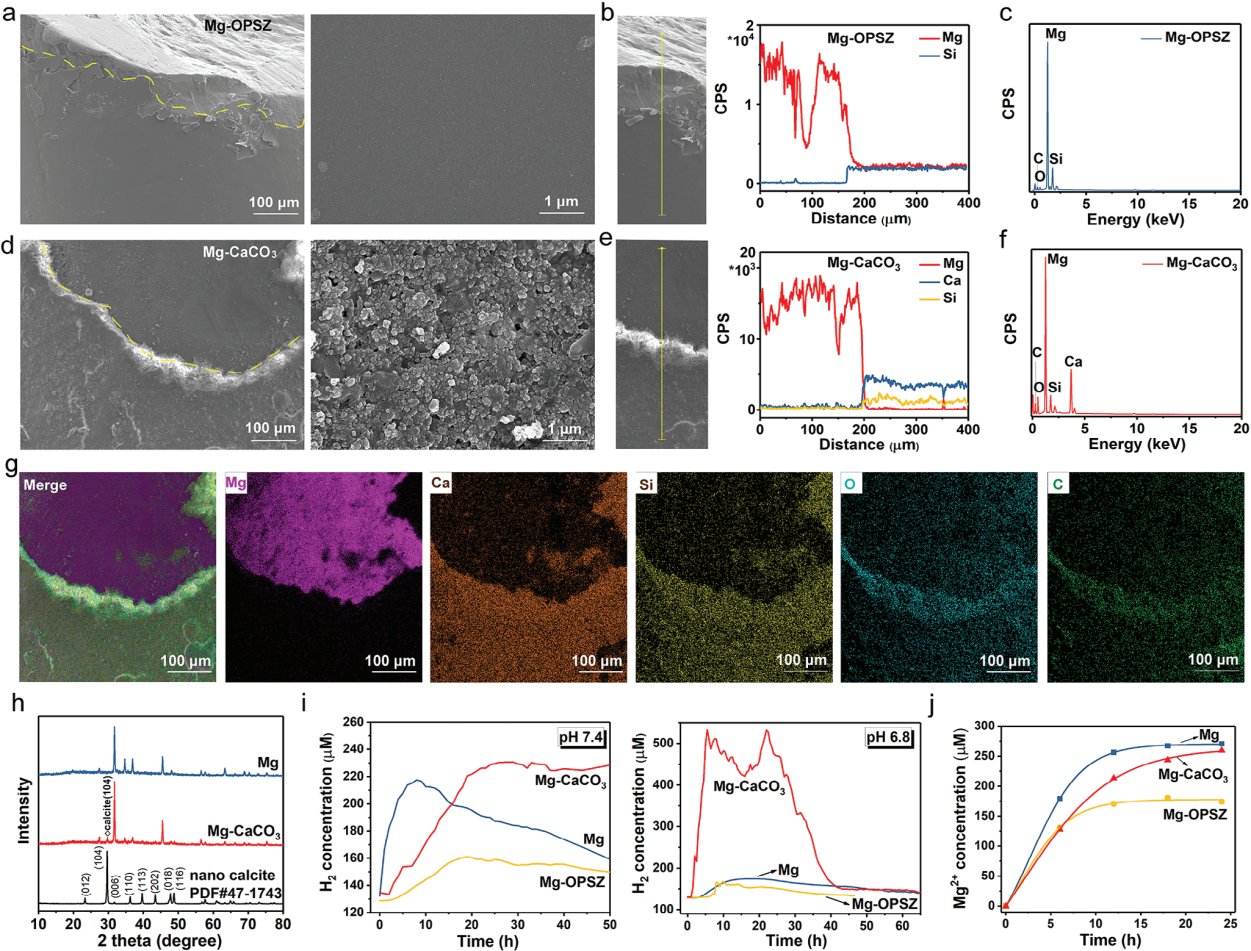
Preparation and characterization of Mg‐CaCO_3_. a,d) The representative SEM images of a) Mg‐OPSZ rods and b) Mg‐CaCO_3_ rods. scale bar: 100 and 1 µm, as indicated. b,e) The topical SEM image and the corresponding distribution of elements in (b) Mg‐OPSZ rods and e) Mg‐CaCO_3_ rods. c,f) EDS spectrum of c) Mg‐OPSZ rods and f) Mg‐CaCO_3_ rods. g) STEM image of the as‐prepared Mg‐CaCO_3_ rods, showing the element distribution of Mg, Ca, Si, O, and C. Scale bar = 100 µm. (h) XRD patterns of Mg rods, Mg‐CaCO_3_ rods, and nano calcite. i) Time‐dependent H_2_ generation measured by hydrogen electrode from Mg, Mg‐OPSZ, or Mg‐CaCO_3_ rods in PBS (pH 7.4 and pH 6.8). j) Mg^2+^ release profiles from Mg, Mg‐OPSZ, or Mg‐CaCO_3_ rods in PBS solutions measured by ICP‐MS.

The Mg‐CaCO_3_ rods were fabricated using a dip‐coating process. Mg wires, roughly 1 or 0.5 mm in diameter, were first polished, washed, and dried. Then, CaCO_3_ nanoparticles (40–80 nm in diameter, Figure S5, Supporting Information) were mixed with organic polysilazane (Durazane 1500SC) in butyl acetate at room temperature to obtain the coating materials. Subsequently, the CaCO_3_/1500SC coating materials were dipped onto Mg wires and cured at 150 °C for 4 h, leading to the formation of Mg‐CaCO_3_ wires. Pure organic polysilazane coating samples without CaCO_3_, referred as Mg‐OPSZ, were also prepared with a similar procedure by changing the dosage of 1500SC. The obtained Mg‐CaCO_3_ and Mg‐OPSZ wires were then cut to rods (4 mm in length) and stored at room temperature for subsequent experiments. The coating and cutting processes covered the majority of the surface area while exposing opening at both ends of the rods. The exposed Mg can react with the surrounding aqueous environment to produce hydrogen bubbles needed for anti‐tumor therapy. Scanning electron microscopy (SEM) imaging was used to visualize the outer morphology of the rods. A distinct interface (yellow dash line) between the Mg matrix and the coating materials was clearly observed at the end of rods (**Figure** **2**a,b). Besides, compared with the smooth surface of the OPSZ coating, CaCO_3_ nanoparticles were clearly visualized on the surface of Mg‐CaCO_3_ (Figure 2b; Figure S5, Supporting Information). Energy‐dispersive X‐ray spectroscopy (EDS) was then used to investigate the chemical mapping of the surface. The element mapping of Mg, Si, and Ca demonstrated the presence of CaCO_3_ and/or OPSZ in the rods (Figure 2c–f). The element mapping of the opening of Mg‐CaCO_3_ rod further confirmed the coating of CaCO_3_ and OPSZ (Figure 2g). The X‐ray diffraction (XRD) analysis showed that the Mg‐CaCO_3_ rod presented the characteristic peak of nano calcite (104), as compared with the Mg rods, confirming the coating of crystalline CaCO_3_ (Figure 2h).

After validating that the Mg‐CaCO_3_ were properly fabricated, we proceeded to evaluate its H_2_ release characteristic in phosphate‐buffered saline (PBS) at pH 7.4. The H_2_ was measured by a hydrogen electrode. As shown in Figure 2i, the Mg rods showed a rapid release of hydrogen in 8 h, followed by a fast decline of hydrogen release in the next 42 h. In contrast, the introduction of OPSZ distinctly decelerate the generation of hydrogen with a maximal amount of 160 µm. Notably, Mg‐CaCO_3_ rods significantly slowed down the rate of hydrogen release, which was mild and the maximal release amount could reach 230 µm, showing a superior hydrogen release capacity to Mg‐OPSZ. In addition, the hydrogen release was evaluated in PBS at pH 6.8, which was more representative of the mildly acidic TME. It was found that the hydrogen release from the Mg‐CaCO_3_ rods was significantly increased, as compared to that in the pH 7.4 PBS. Moreover, the maximal hydrogen release of Mg‐CaCO_3_ rods in pH 7.4 PBS could reach 500 µm. However, in contrast, the hydrogen release of Mg‐OPSZ rods showed no difference in pH 7.4 PBS and pH 6.8 PBS. Since CaCO_3_ nanoparticles have been thoroughly studied as pH‐responsive theranostic agents with excellent biocompatibility and biodegradability, we speculated that the CaCO_3_ nanoparticles endowed the Mg‐CaCO_3_ rods with pH responsive hydrogen release capacity. Therefore, accompanied with evaluations of corrosion and H_2_ release, Mg ions and Ca ions release were also evaluated. The Mg ions concentration released from both Mg‐OPSZ and Mg‐CaCO_3_ rods was below 250 µm and the Ca ions concentration released from the Mg‐CaCO_3_ rods was ca. 60 µm after 24 h, as determined by the inductively coupled plasma‐mass spectrometry (ICP‐MS) (Figure 2j; Figure S6, Supporting Information).

### 2.3 Hydrogen Therapy by Mg‐CaCO_3_ Kills Tumor Cells through Redox Homeostasis Disruption

**Figure 3:**
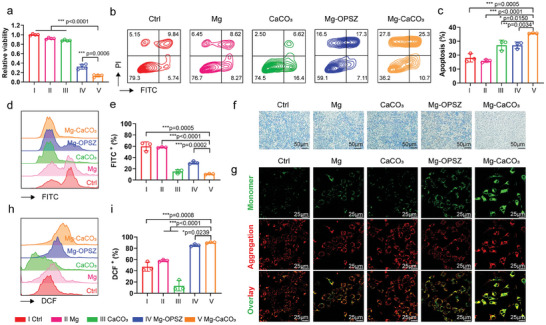
In vitro evaluation of the anti‐tumor effects of Mg‐CaCO_3_. a) Relative cell viabilities of 4T1 cells with different treatments for 24 h (*n* = 4). b,c) Flow cytometry assay (b) and corresponding quantitative analysis (c) of the extent of 4T1 cells apoptosis by Annexin V/PI staining (*n* = 3). d,e) Flow cytometry assay (d) and corresponding quantitative analysis (e) of pH level in 4T1 cells by the BCECF AM probe (*n* = 3). f) Qualitative analysis of hydrogen generation in MB‐stained 4T1 cells. Scale bar = 50 µm. g) Fluorescence microscope images of 4T1 cells with different treatments and then stained with JC‐1 dye. Scale bar = 25 µm. h,i) Flow cytometry assay (h) and corresponding quantitative analysis (i) of intracellular ROS level (DCF^+^ cell ratio) in 4T1 cells by DCFH‐DA probe (*n* = 3). Group: I: Ctrl, II: Mg, III: CaCO_3_, IV: Mg‐OPSZ, V: Mg‐CaCO_3_. Statistical significance was calculated via two‐tailed Students’ *t*‐test. ^*^ *p* < 0.05, ^**^ *p* < 0.01, and ^***^ *p* < 0.001. The mean values and SD are presented.

Before the application of Mg‐CaCO_3_ in anti‐tumor therapy, we first estimated its biological effects on tumor cells. For in vitro experiments, we used Mg^2+^ (equal to the end‐point Mg^2+^ concentration of Mg‐CaCO_3_) as Mg‐control group (labeled as II: Mg) and CaCO_3_ nanoparticles (equal to the concentration used for Mg‐CaCO_3_ modification) as CaCO_3_‐control group (labeled as III: CaCO_3_). Also, blank control was labeled as I: Ctrl, Mg rod with OPSZ modification was labeled as IV: Mg‐OPSZ group, and Mg rod with CaCO_3_ modification was labeled as V: Mg‐CaCO_3_ group. Interestingly, although we designed Mg‐CaCO_3_ based on the purpose of remodeling CAFs and TME, Mg‐CaCO_3_ itself did exhibit remarkable anti‐tumor capability. As indicated by CCK8 assay, both Mg‐OPSZ and Mg‐CaCO_3_ inhibited 4T1 cell viability, and Mg‐CaCO_3_ (with a higher and more stable hydrogen concentration) showed better anti‐tumor capability than Mg‐OPSZ (IV: 0.32, V: 0.13) (**Figure** **3**a). In order to exclude irrelevant factors, we also evaluated 4T1 cell viability with different Mg^2+^, CaCO_3_ concentration, and pH levels in culture medium. No statistical significance was found between treatment groups (Figure S7, Supporting Information). Besides, 4T1 cells treated by Mg‐CaCO_3_ exhibited higher apoptosis ratio (I: 18.12, II: 15.91, III: 27.18, IV: 27.14, V: 35.70) and generated higher calreticulin (CRT) (I: 32.80, II: 31.13, III: 55.57, IV: 84.47, V: 83.73), compared to other four groups (Figure 3b,c; Figure S8a–c, Supporting Information).

With the abovementioned significant anti‐tumor ability, we further evaluated the underlying mechanism. First, we used a pH probe (BCECF AM probe) to indicate intracellular pH after different treatments. The BCECF AM probe shows stronger green fluorescent with lower pH value, which could be analyzed by flow cytometry assay (Figure 3d,e).^[^ ^27^ ^]^ As expected, Ctrl and Mg groups exhibited relatively low pH, and both CaCO_3_ and Mg‐CaCO_3_ group showed decreased FITC^+^ cell ratio (III: 15.13, V: 10.57), indicating an up‐regulation of intracellular pH in these two groups. These results demonstrated that CaCO_3_ modification endowed Mg‐CaCO_3_ with good pH‐response capability. Then, we used methylene blue (MB), which can be reduced to colorless leucomethylene blue by the hydrogen, as a probe for rapid detection of hydrogen gas.^[^ ^28^ ^]^ As shown in Figure 3f, tumor cells exhibited blue color after co‐incubating with the MB probe. Compared with the Ctrl group, the blue color was not significantly reduced in Mg or CaCO_3_ groups since there was no hydrogen generation. The blue color was reduced after the Mg‐OPSZ treatment while the change was more prominent with the Mg‐CaCO_3_ treatment, implying efficient hydrogen generation within cancer cells (Figure 3f; Figure S8d, Supporting Information). According to previous reports, hydrogen gas can induce cancer cell death by inducing mitochondrial dysfunction, which is often accompanied by the decrease in mitochondrial membrane potential (MMP).^[^ ^29^ ^]^ Tetrechloro‐tetraethyl benzimidazol carbocyanine iodide (JC‐1), a commonly used fluorescent indicator, was employed to measure MMP changes. When the MMP is high, JC‐1 will form aggregates with red fluorescence in the mitochondrial matrix. On the contrary, when the MMP is low, JC‐1 will exist as monomers with green fluorescence. In this way, the change of MMP can be easily detected by the change of fluorescence color. As showed in Figure 3g, the Mg‐CaCO_3_ group displayed obvious green fluorescence, demonstrating that the generated hydrogen by Mg‐CaCO_3_ effectively damaged the mitochondria of 4T1 cells. Next we used 2′, 7′‐dichlorodihydrofluorescein diacetate (DCFH‐DA) as a classical probe to monitor changes in ROS levels.^[^ ^30^ ^]^ By flow cytometry analysis, a significant upregulation of intracellular ROS (DCF^+^ cell ratio) was observed in the Mg‐CaCO_3_ group (I: 47.20, II: 58.00, III: 13.32, IV: 85.33, V: 90.13) (Figure 3h,i; Figure S8e, Supporting Information). These results collectively revealed that Mg‐CaCO_3_ could induce cancer cell death through hydrogen‐induced mitochondrial damage and redox homeostasis disruption.

### 2.4 Hydrogen Therapy by Mg‐CaCO_3_ Remodels CAFs’ Immune‐Suppressive Phenotypes

**Figure 4:**
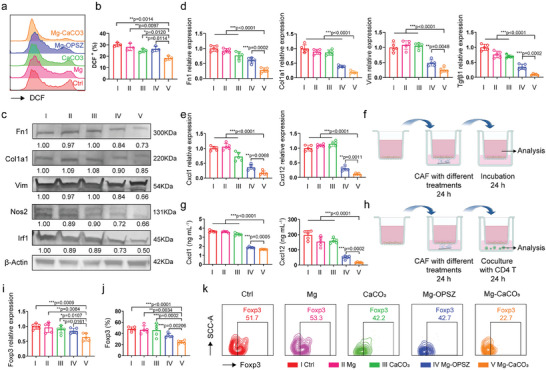
In vitro evaluation of the CAF remodeling and immune‐modulatory effects of Mg‐CaCO_3_. a,b) Flow cytometry assay (a) and corresponding quantitative analysis (b) of intracellular ROS level (DCF^+^ cell ratio) in CAFs with different treatments (*n* = 3). c) Western blot analysis of CAF marker proteins of CAFs with different treatments. d) MRNA levels of CAF markers analyzed by qPCR in CAFs with different treatments (*n* = 5). e) MRNA levels of *Cxcl1* and *Cxcl12* analyzed by qPCR in CAFs with different treatments (*n* = 5). f,h) Schematic illustration of the in vitro co‐culture model using transwell f) without and h) with CD4^+^ T cells (created with bioRender.com). g) Quantification of the concentration of Cxcl1 and Cxcl12 levels in the supernatant of CAFs with different treatments (*n* = 5). i) MRNA levels of *Foxp3* analyzed by qPCR in CD4^+^ T cells co‐cultured with CAFs with different treatments (*n* = 5). j,k) Flow cytometry assay (k) and corresponding quantitative analysis (j) for Treg (Foxp3^+^ cell) in CD4^+^ T cells co‐cultured with CAFs with different treatments (*n* = 5). Group: I: Ctrl, II: Mg, III: CaCO_3_, IV: Mg‐OPSZ, V: Mg‐CaCO_3_. Statistical significance was calculated via two‐tailed Students’ *t*‐test. ^*^ *p* < 0.05, ^**^ *p* < 0.01, and ^***^ *p* < 0.001. The mean values and SD are presented.

Next, we focused on biological effects of Mg‐CaCO_3_ on CAFs and evaluated whether it could reverse the CAFs’ phenotypes as observed in the hydrogen treatment model. As showed in Figure S9a (Supporting Information), unlike the potent cytotoxic effects on tumor cells, Mg‐CaCO_3_ treatment killed fewer CAFs (relative viability of group V: 0.7802), suggesting it did not kill CAFs directly and had a different biological influence on CAFs. By JC‐1 dye staining assay, we observed weak green fluorescence in the Ctrl group, while the green fluorescence further decreased in Mg‐OPSZ and Mg‐CaCO_3_ group (Figure S9b, Supporting Information). By DCFH‐DA probe staining assay, a down‐regulation of intracellular ROS level (DCF^+^ cell ratio) was observed in the Mg‐CaCO_3_ group (I: 30.33, II: 28.20, III: 24.93, IV: 26.43, V: 18.50) (**Figure** **4**a,b). Since tumor cells and CAFs exhibited totally different intracellular metabolic and biological procession,^[^ ^31^ ^]^ these results indicated that hydrogen therapy by Mg‐CaCO_3_ could decrease MMP and alleviate ROS within CAFs. As mentioned above, *Nos2* was a stress‐induced gene, which could be up‐regulated by ROS signal and then regulate down‐stream pro‐tumor factors expression.^[^ ^26^ ^]^ We confirmed that the expression levels of Nos2 and its reported transcription factor (TF) Irf1 significantly decreased after the Mg‐OPSZ and Mg‐CaCO_3_ treatment,^[^ ^32^ ^]^ while Mg‐CaCO_3_ showed a better inhibition effect (Figure 4c). Concomitant with the down‐regulation of *Nos2*, multiple CAF markers,^[^ ^24^, ^33^ ^]^ including *Fn1*, *Col1a1*, *Vim*, *α‐Sma*, and *Tgf‐β1*, were also detected to be down‐regulated in the Mg‐CaCO_3_ group by qPCR, Western blot, and immunocytochemistry assays (Figure 4c,d; Figure S10, Supporting Information). These results suggested that the CAFs’ phenotypes were reversed by the hydrogen therapy by Mg‐CaCO_3_.

According to current studies, CAFs could produce various pro‐tumor factors, such as matrix metalloproteinases (Mmp) 2 (Mmp2), and Il‐6 to enhance tumor migration, invasion, and drug‐resistance and to generate an immune‐suppressive TME.^[^ ^34^, ^35^ ^]^ We examined expression levels of these genes in CAFs after different treatments and observed a widely expression inhibition of these pro‐tumor factors (Figure S10a, Supporting Information). Notably, among these pro‐tumor factors, we found two secreted chemokine genes, *Cxcl1* and *Cxcl12*, were consistently down‐regulated in the Mg‐CaCO_3_ group (relative expression level of group V: *Cxcl1*: 0.1757, *Cxcl12*: 0.1154) (Figure 4e). Since Cxcl1 and Cxcl12 are secreted factors, we also used co‐culture model and collected cell supernatant for ELISA analysis and found significantly decreased Cxcl1 (I: 3.646, II: 3.590, III: 3.321, IV: 1.872, V: 1.664) and Cxcl12 (I: 197.1, II: 154.3, III: 158.5, IV: 50.16, V: 16.47) concentrations in the Mg‐CaCO_3_ group (Figure 4f,g). Reviewing current studies, Cxcl1 and Cxcl12 could both generate CD4^+^ T cells to an immune‐suppressive phenotype and are associated with Treg infiltration,^[^ ^36^, ^37^, ^38^ ^]^ which was also seen in our in silico analysis (Figure 1c). Therefore, we further included CD4^+^ T cells in the co‐culture model and then evaluated CD4^+^ T cell phenotypes (Figure 4h). Briefly, CAFs were seeded in the upper chamber and after different treatments for 24 h, the upper chamber was put into another well and co‐cultured with mice CD4^+^ T cells. By both qPCR and flow cytometry analysis, we observed lower *Foxp3* expression level (I: 1.000, II: 0.9640, III: 0.9023, IV: 0.8500, V: 0.6369) as well as lower Treg ratio (I: 47.90, II: 46.04, III: 46.68, IV: 36.22, V: 24.56) in the Mg‐CaCO_3_ group (Figure 4i–k). In the meantime, we also detected higher immune activating biomarkers (*Nkg7* and ICOS) and lower immune suppressive biomarker (*Lag3*) in the Mg‐CaCO_3_ group (Figure S11, Supporting Information). These results showed that hydrogen therapy by Mg‐CaCO_3_ alleviated ROS in CAFs and inhibited *Nos2* expression, which induced CAFs’ phenotypes reversion and further CD4^+^ T cell stimulation.

### 2.5 In Vivo Safety and Anti‐Tumor Effects of Hydrogen Therapy by Mg‐CaCO_3_

**Figure 5:**
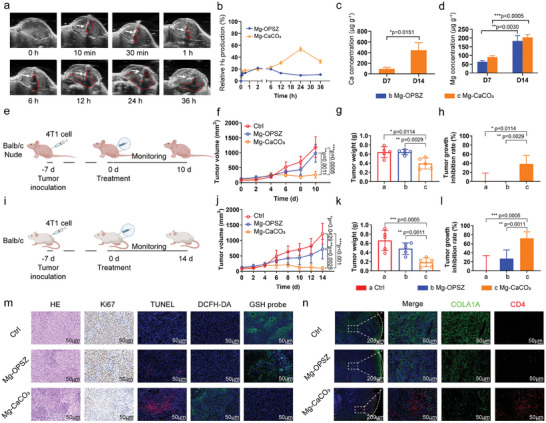
In vivo anti‐tumor and TME remodeling effects of Mg‐CaCO_3_. a) In vivo time‐dependent ultrasonic imaging of 4T1 tumor‐bearing mice after intratumoral administration with Mg‐OPSZ and Mg‐CaCO_3_ rods. b) Quantitative analysis of signal intensities based on ultrasonic imaging data. c,d) The Ca (c) and Mg (d) levels in tumors of mice after different treatments for 7 days and 14 days (*n* = 3). e,i) Schematic illustration of subcutaneous 4T1 mouse tumor model in Balb/c nude (e) and Balb/c (i) mice (created with bioRender.com). f–h) Tumor growth curves (f), tumor weight (g), and tumor inhibition rate (h) of Balb/c nude mice after different treatments (*n* = 5). j–l) Tumor growth curves (j), tumor weight (k), and tumor inhibition rate (l) of Balb/c nude mice after different treatments (*n* = 5). m) Microscopy images of H&E, Ki67, TUNEL, DCFH‐DA, and GSH probe staining of tumors collected from Balb/c mice after different treatments. Scale bar = 50 µm. n) IF images of tumors collected from Balb/c mice after different treatments. Green: COL1A1, red: CD4, blue: DAPI, scale bar: 200 and 50 µm, as indicated. Group: a: Ctrl, b: Mg‐OPSZ, c: Mg‐CaCO_3_. Statistical significance was calculated via two‐tailed Students’ *t*‐test. ^*^ *p* < 0.05, ^**^ *p* < 0.01, and ^***^ *p* < 0.001. The mean values and SD are presented.

Next, we applied Mg‐CaCO_3_ for in vivo anti‐tumor therapy. We set blank control (Ctrl group), Mg‐OPSZ group, and Mg‐CaCO_3_ group for in vivo experiments (excluding Mg^2+^ and CaCO_3_ groups for the impracticability). First, ultrasonic imaging was performed to monitor hydrogen gas generation in the tumor, as gas bubbles would offer strong contrast under ultrasound imaging (**Figure** **5**a,b). The ultrasound signals in the Mg‐OPSZ group were weak, indicating the insignificant hydrogen generation from the Mg‐OPSZ. In contrast, after Mg‐CaCO_3_ was implanted into the tumor, strong ultrasound signals appeared in the tumor for 36 h, suggesting efficient and stable gas production capability of Mg‐CaCO_3_ and the superiority of Mg‐CaCO_3_ for TME‐responsive H_2_ generation. Then, in order to further characterize bio‐degradation behaviors of Mg‐CaCO_3_, the Ca and Mg levels in the tumors of mice after being implanted with rods for different times were measured by ICP‐MS (Figure 5c,d). For Mg‐OPSZ, the intratumoral Mg concentration increased in day 14 compared to day 7 (day 7: 63.78, day 14: 182.38). Similarly, for Mg‐CaCO_3_, the Ca (day 7: 96.47, day 14: 444.34) and Mg (day 7: 89.968, day 14: 202.79) concentration in the tumor gradually increased over time, which indicated a continuing degradation of Mg‐OPSZ and Mg‐CaCO_3_. However, there was no significant increase of Ca and Mg levels in the blood and major organs after implantation, indicating that Ca^2+^ and Mg^2+^ generated from the degraded Mg‐CaCO_3_ would not affect the equilibrium of Ca and Mg levels in the whole body (Figure S12, Supporting Information). In the meantime, we evaluated other blood indicators to check whether there were potential toxic side effects. For the Mg‐CaCO_3_ group, alanine aminotransferase (ALT), aspartic aminotransferase (AST), creatinine (CREA), and uric acid (UA), all of which were important liver and kidney function markers, were within the reference range and were comparable to the control group on day 7 and day 14, indicating that Mg‐CaCO_3_ implanted caused no significant hepatotoxicity or nephrotoxicity (Figure S12c, Supporting Information). Then, the hematological assessment, including white blood cells (WBC), red blood cells (RBC), hemoglobin (HGB), hematocrit (HCT), and mean corpuscular hemoglobin (MCH) were found to be normal in the Mg‐CaCO_3_ group compared to that in the Ctrl group on both day 7 and day 14 (Figure S12c, Supporting Information). Meanwhile, no obvious histological damage was observed on organ slices from Mg‐CaCO_3_ treated mice (Figure S13, Supporting Information) and the body weight fluctuation curves showed no significant differences between these groups (Figure S14, Supporting Information).

Based on these satisfactory biosafety results, we next carefully investigated the anti‐tumor efficacy of hydrogen therapy by Mg‐CaCO_3_. Balb/c nude mice were our first choice because they had deficient immune systems and thus transplanted tumors were able to grow rapidly in them. Mice bearing subcutaneous 4T1 tumors were randomly divided into three groups (*n* = 5): a) Ctrl group, b) Mg‐OPSZ group, and c) Mg‐CaCO_3_ group. For Mg‐OPSZ and Mg‐CaCO_3_ groups, Mg‐OPSZ or Mg‐CaCO_3_ rods were implanted into each tumor (Figure 5e). The tumors of Mg‐OPSZ group showed slightly delayed growth. Remarkably, the tumor growth of Mg‐CaCO_3_ group was significantly inhibited (Figure 5f; Figure S15, Supporting Information). The smallest tumor weight and highest inhibition rate were observed in the Mg‐CaCO_3_ group (Figure 5g,h). We then collected tumor slices for staining analysis. Hematoxylin and eosin (H&E) staining, Ki67 staining, and TdT‐mediated dUTP nick‐end labeling (TUNEL) staining of tumor slices confirmed that Mg‐CaCO_3_ implantation could induce severe tumor cell apoptosis (Figure S16, Supporting Information). Notably, in order to characterize biological effects of hydrogen treatment, we used DCFH‐DA probe to evaluate ROS levels and ThiolTracker violet dye to evaluate consumption of intratumoral GSH in different groups. As revealed by immunofluorescence (IF) microscopy, the Mg‐CaCO_3_ group exhibited stronger green fluorescence in DCFH‐DA staining and weaker green fluorescence in GSH probe staining than the other two groups, indicating hydrogen therapy could induce ROS accumulation within tumors and oxidize GSH to GSSG in cancer cells (Figure S16, Supporting Information). In the meantime, we conducted IF staining to characterize CAF distribution in different groups and found that green fluorescence was weaker and more dispersedly distributed within tumors in the Mg‐CaCO_3_ group (Figure S17, Supporting Information). These results revealed that hydrogen therapy by Mg‐CaCO_3_ could directly kill tumor cells and also affect CAFs in the TME.

In order to further evaluate CAFs regulating and TME remodeling effects of hydrogen therapy by Mg‐CaCO_3_, we used immune competent mice Balb/c for in vivo experiments (Figure 5i). Consistent with results in nude mice, tumors in the Mg‐CaCO_3_ group showed a most inhibited growth curve among three groups (Figure 5j; Figure S18, Supporting Information). Notably, as for tumor weight and tumor growth inhibition rate, hydrogen therapy by Mg‐CaCO_3_ showed a superior anti‐tumor efficacy in immune competent mice (tumor growth inhibition rate: nude: 38.47, balb/c: 71.98), suggesting that the efficiency of hydrogen therapy was associated with the immune microenvironment. Then by immunohistology (IHC) and IF staining, we further delineated TME remodeling after Mg‐CaCO_3_ implantation (Figure 5m,n; Figure S19, Supporting Information). In the Mg‐CaCO_3_ group, an obvious down‐regulation of NOS2 fluorescence was observed in CAFs, consistent with our in vitro analysis. Moreover, the slices were co‐stained with COL1A1 and CD4 to characterize the relation between CAFs and CD4^+^ T cells. We found that in Ctrl and Mg‐OPSZ tumors, CAFs formed tumor capsules and were widely distributed in the inner part, excluded CD4^+^ T cells from efficiently infiltration. However, upon Mg‐CaCO_3_ implantation, CAFs became loosen with a closer localization of CD4^+^ T cells. These results collectively demonstrated that hydrogen therapy by Mg‐CaCO_3_ could remodel CAFs’ phenotypes for immune infiltration.

### 2.6 Cold Tumor Remodeling and Systematic Immune Stimulating Effects of Hydrogen Therapy by Mg‐CaCO_3_

**Figure 6:**
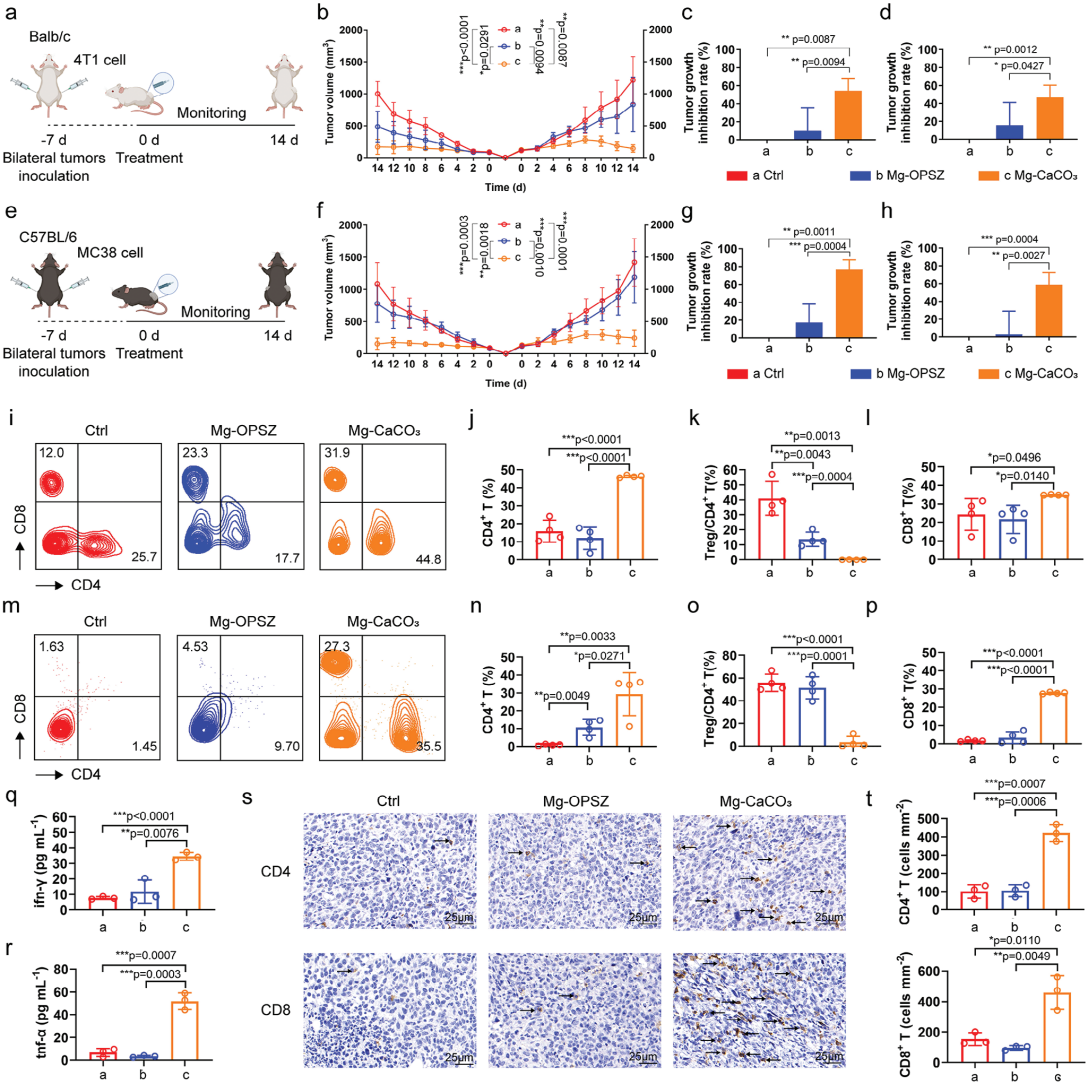
Systematic immune activating effects of Mg‐CaCO_3_. a,e) Schematic illustration of bilateral tumor transplantation model of 4T1 in Balb/c (a) and MC38 in C57BL/6 (e) mice (created with bioRender.com). b–d) Tumor growth curves (b) and tumor inhibition rate of primary tumors (c) and distant tumors (d) of Balb/c mice after different treatments (*n* = 5). f–h) Tumor growth curves (f) and tumor inhibition rate of primary tumors (g) and distant tumors (h) of C57BL/6 mice after different treatments (*n* = 5). i) Flow cytometry assay of CD4^+^ and CD8^+^ T cells in primary tumors collected from C57BL/6 mice after different treatments. j–l) Quantification of j) CD4^+^/CD45^+^ ratio, k) Treg/CD4^+^ ratio, and l) CD8^+^/CD45^+^ ratio related to (i) (n = 4). m) Flow cytometry assay of CD4^+^ and CD8^+^ T cells in primary tumors collected from Balb/c mice after different treatments. n–p) Quantification of CD4^+^/CD45^+^ ratio (n), Treg/CD4^+^ ratio (o), and CD8^+^/CD45^+^ ratio (p) related to (m) (n = 4). q,r) Quantification of ifn‐γ (q) and tnf‐α (r) concentration in the serum of Balb/c mice after different treatments (*n* = 3). s) Representative IHC images of CD4^+^ and CD8^+^ T cell infiltration in distant tumors collected from Balb/c mice after different treatments. Scale bar = 25 µm. t) Quantification analysis of CD4^+^ and CD8^+^ T cell infiltration related to (s) (*n* = 3). Group: a: Ctrl, b: Mg‐OPSZ, c: Mg‐CaCO_3_. Statistical significance was calculated via two‐tailed Students’ *t*‐test. ^*^ *p* < 0.05, ^**^ *p* < 0.01, and ^***^ *p* < 0.001. The mean values and SD are presented.

Inspired by the immune stimulating effects of Mg‐CaCO_3_, we then evaluated its systematic immune stimulating effects by the bilateral tumor inoculation model (**Figure** **6**a,e). Here, we chose two different tumor models, including murine breast cancer 4T1 cells and murine colon cancer MC38 cells. 4T1 cell model was considered as “cold tumor” with relatively low immune infiltration while MC38 cell model was thought to be “hot tumor” with high level of immune reaction.^[^ ^39^, ^40^ ^]^ We compared results of these two models to evaluate therapy efficacy of Mg‐CaCO_3_ on mice with different immune baseline levels. First, the overall tumor inhibition effects of Mg‐CaCO_3_ were similar in 4T1 and MC38 cell models. In the 4T1 cell model, the best suppression effect of the primary tumors was observed in the Mg‐CaCO_3_ group with 54.34% tumor growth inhibition rate (Figure 6b,c; Figure S20a–c, Supporting Information), which was slightly lower than that in the unilateral tumor inoculation model and may be due to different tumor burdens in these two models. For distant tumors, we observed that the suppression effects of Mg‐CaCO_3_ were significantly superior to Mg‐OPSZ (Mg‐OPSZ: 15.78, Mg‐CaCO_3_: 46.69), suggesting the hydrogen therapy could not only inhibit tumors in situ, but also present a systematic anti‐tumor effects (Figure 6d; Figure S20d–f, Supporting Information). The H&E, Ki67, TUNEL, DCFH‐DA, and GSH probe staining and IF staining with anti‐NOS2 antibody confirmed CAF remodel effects and tumor suppression of both primary and distant tumors (Figures S21 and S22, Supporting Information). In MC38 cell models, we also observed the satisfactory tumor suppression efficacy in both primary and distant tumors (Figure 6f–h; Figure S23, Supporting Information). As for specific tumor growth inhibition rates, those in MC38 tumor model were superior to their 4T1 model counterparts (Mg‐CaCO_3_ in primary tumors: 4T1:54.34, MC38: 77.15; Mg‐CaCO_3_ in distant tumors: 4T1: 46.69, MC38: 58.81). The H&E, Ki67, TUNEL, DCFH‐DA, and GSH probe staining and IF staining with anti‐NOS2 antibody obtained consistent results with those in 4T1 cells (Figures S24 and S25, Supporting Information).

As for anti‐tumor immunity evaluation, by flow cytometry, we observed an overall higher immune cell infiltration level in the Ctrl group in MC38 tumor models than that in 4T1 models (4T1: CD4^+^ T: 0.9450, CD8^+^ T: 1.695; MC38: CD4^+^ T: 15.88, CD8^+^ T: 24.33), indicating their activated immune status in the baseline (Figure 6i–p; Figures S26 and S27, Supporting Information). When comparing T cell infiltration ratio, up‐regulation of both CD4^+^ T and CD8^+^ T cell infiltration were observed in the Mg‐CaCO_3_ group in the MC38 tumor model (CD4^+^ T: Ctrl: 15.88, Mg‐OPSZ: 11.95, Mg‐CaCO_3_: 46.33; CD8^+^ T: Ctrl: 24.33, Mg‐OPSZ: 21.63, Mg‐CaCO_3_: 34.85) with a significant decreased Treg ratio (Ctrl: 40.93, Mg‐OPSZ: 13.63, Mg‐CaCO_3_: 0.1875) (Figure 6i–l). More importantly, in the 4T1 cell model, immune infiltration exhibited more drastic increases after therapy (CD4^+^ T: Ctrl: 0.9450, Mg‐OPSZ: 10.67, Mg‐CaCO_3_: 29.30; CD8^+^ T: Ctrl: 1.695, Mg‐OPSZ: 3.3888, Mg‐CaCO_3_: 27.78), as the CD4^+^ and CD8^+^ T ratio values of Mg‐CaCO_3_ group were comparable to those in the MC38 cell model (Figure 6m–p). These results supported the capability of Mg‐CaCO_3_ to remodel immune microenvironment, enhance immune infiltration, and turn the “cold tumor” into “hot tumor”.

Moreover, the immune stimulating factors ifn‐γ and tnf‐α in the serum are key elements for anti‐tumor efficiency.^[^ ^41^, ^42^ ^]^ We found a higher level of ifn‐γ and tnf‐α in the Ctrl group in MC38 cell model than 4T1 cell model (4T1: ifn‐γ: 7.736, tnf‐α: 6.780; MC38: ifn‐γ: 20.96, tnf‐α: 17.73) (Figure 6q,r; Figure S28, Supporting Information), which further supported an activated immune status of MC38 cell model. However, after hydrogen therapy by Mg‐CaCO_3_, both ifn‐γ and tnf‐α in mice serum distinctly increased in the 4T1 cell model (ifn‐γ: Ctrl: 7.736, Mg‐OPSZ: 25.92, Mg‐CaCO_3_: 34.56; tnf‐α: Ctrl: 6.780, Mg‐OPSZ: 3.320, Mg‐CaCO_3_: 51.91) (Figure 6q,r) and the MC38 cell model (ifn‐γ: Ctrl: 20.96, Mg‐OPSZ: 17.34, Mg‐CaCO_3_: 42.54; tnf‐α: Ctrl: 17.73, Mg‐OPSZ: 23.95, Mg‐CaCO_3_: 54.69) (Figure S28, Supporting Information), confirming the activation of systematic anti‐tumor immunity. Finally, by IHC analysis of the distant tumors, we found the up‐regulation of both CD4^+^ and CD8^+^ T cell infiltration in the MC38 distant tumors (CD4^+^ T: Ctrl: 190.3, Mg‐OPSZ: 246.0, Mg‐CaCO_3_: 384.0; CD8^+^ T: Ctrl: 232.7, Mg‐OPSZ: 369.3, Mg‐CaCO_3_: 811.7) (Figure S29, Supporting Information). As for 4T1 distant tumors, the immune infiltration level was up‐regulated to a level similar to that of MC38 tumors (CD4^+^ T: Ctrl: 100.7, Mg‐OPSZ: 104.7, Mg‐CaCO_3_: 421.7; CD8^+^ T: Ctrl: 153.7, Mg‐OPSZ: 96.67, Mg‐CaCO_3_: 458.7) (Figure 6s,t). Considering the immune stimulation effects of Mg‐CaCO_3_ therapy, we also investigated whether it has a synergistic effect with immune checkpoint inhibitors (ICIs). The Mg‐CaCO_3_ was implanted intratumorally on day 0, followed by intraperitoneal injection with andibody against PD1 (αPD1) on day 1, 3, and 5 (Figure S30a, Supporting Information). It was clear that the combination treatment with Mg‐CaCO_3_ and αPD1 remarkably promoted the tumor suppression effect, as compared with the αPD1 monotherapy. Notably, all mice in the control group died within 40 days while mice after the combination treatment exhibited a 100% survival within 60 days (Figure S30b,c, Supporting Information).

Collectively, these results confirmed the TME remodeling and systematic immune activation effects of Mg‐CaCO_3_ therapy, especially for those “cold tumors” with deficient immune infiltration.

## 3 Discussion

In the current study, we observed that the hydrogen treatment could reverse immune suppressive and pro‐tumor phenotypes of CAFs. CAFs are important components of the TME, which could secrete a variety of growth factors and chemokines to promote tumor growth and induce immune suppression.^[^ ^24^ ^]^ Although previous studies on hydrogen therapy reported that hydrogen could directly kill tumor cells by inducing mitochondrial dysfunction and intracellular redox homeostasis destruction,^[^ ^6^, ^10^ ^]^ hydrogen's biological effects on CAFs lack sufficient reports yet. Interestingly, in our study, we found that hydrogen treatment actually alleviated ROS in CAFs and down‐regulated a key ROS‐induced gene, *Nos2*, and down‐stream pro‐tumor factors such as *Mmp2*, *Cxcl1*, and *Cxcl12*. This significant biological mechanism difference of hydrogen gas on tumor cells and CAFs may be due to the metabolic and redox balance differences between these two cell types.^[^ ^43^ ^]^ Based on this discovery, we designed hydrogen‐producing Mg rods. In order to achieve rapid, stable, and TME‐responsive hydrogen release effect, CaCO_3_ nanoparticles were used for surface modification (Mg‐CaCO_3_). Given the weak acid environment of tumors,^[^ ^44^ ^]^ CaCO_3_ could be dissolved continuously and enhance Mg alloy to release hydrogen gas. By both in vitro and in vivo hydrogen release models, we observed a superior hydrogen release capability of Mg‐CaCO_3_ compared to Mg or Mg‐OPSZ rods.

Then, we evaluated the anti‐tumor biological effects of hydrogen therapy by Mg‐CaCO_3_. We found that Mg‐CaCO_3_ could directly kill tumor cells and inhibit tumor growth in mice tumor transplantation models, which is consistent with previous reported hydrogen therapies.^[^ ^6^, ^10^, ^11^, ^45^ ^]^ Moreover, we observed that CAFs significantly down‐regulated a series of pro‐tumor factors and immune suppression chemokines including Cxcl1 and Cxcl12 after Mg‐CaCO_3_ treatment. These two chemokines are reported to be associated with Treg differentiation. And in further evaluation, we found the inhibition of immune suppressive phenotype transition of CD4^+^ T cells in the Mg‐CaCO_3_ group. When reviewing current hydrogen therapies, they only focused on tumor cells themselves, but paid little attention to TME components and the association between stromal microenvironment and immune microenvironment. Therefore, in our study, we delineated the therapy effects of Mg‐CaCO_3_ on stromal and immune microenvironment thoroughly. As for in vivo application, we compared immune‐deficient mice with immune‐competent mice and found that the latter showed better therapeutic efficacy, which indicated that the CAFs’ phenotypes reversing and immune activation effects played an important role in the hydrogen therapy by Mg‐CaCO_3_. Moreover, we compared therapy effects of a highly immunogenic MC38 cell model (“hot tumor”) with a low immune‐infiltrated 4T1 cell model (“cold tumor”). We found that although MC38 cell tumor model showed higher immune infiltration level than 4T1 cell model in the primary tumor at baseline, after Mg‐CaCO_3_ implantation, the TME of 4T1 cell tumor could be remodeled to become “hot”, with significantly up‐regulated immune infiltration to the level of MC38 cell tumors. In the meantime, immune factor secretion and immune infiltration in the distant tumors were up‐regulated, suggesting Mg‐CaCO_3_ generated comprehensive activation effects on systematic immunity. Finally, we investigated the synergistic effect of Mg‐CaCO_3_ combining with ICIs and found that combining with Mg‐CaCO_3_ could enhance therapy efficancy of αPD1 and achieved longer survivals. These results indicated that the Mg‐CaCO_3_ therapy had the potent TME remodeling and systematic immune activation effects, especially for those “cold tumors” with deficient immune infiltration.

The major limitation of Mg‐CaCO_3_ therapeutic is the inapplicability for tumors undergoing surgery. However, given the superior TME‐remodeling and immunostimulating capability, we supposed that Mg‐CaCO_3_ could be applied for treating unresectable tumors, especially “cold tumors”. Moreover, though we have confirmed the biosafety and compatibility of Mg‐CaCO_3_ by blood biochemistry assay and histological evaluation of major organs, when applying Mg‐CaCO_3_ rods to patients clinically, the safety issue should be further evaluated.

## 4 Conclusion

**Figure 7:**
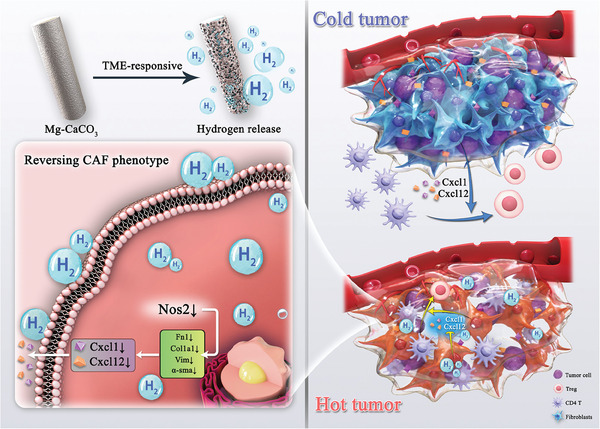
Schematic illustration of TME remodeling and tumor inhibition mechanisms of Mg‐CaCO_3_. The TME‐responsive Mg‐CaCO_3_ rods reverse CAFs’ phenotypes, down‐regulate immune suppressive factors secretion in CAFs, remodel the “cold tumor” to the “hot tumor”, and activate effective systematic immunity.

In summary, we found that hydrogen could reverse CAFs’ phenotypes and we developed a TME‐responsive Mg‐CaCO_3_ rods for in situ hydrogen therapy. The hydrogen therapy by Mg‐CaCO_3_ could not only kill tumor cells, but also down‐regulate pro‐tumor and immune suppressive factors in CAFs. In this way, we were able to turn the “cold tumor” into the “hot tumor”, with higher CD4^+^ T and CD8^+^ T cells infiltration, higher immune stimulating factors secretion, and an effective systematic immune activation (**Figure** **7**).

## 5 Experimental Section

### Study Approval

All animal experiments were performed in accordance with the Guide for the Care and Use of Laboratory Animals (China, GB/T 35892‐2018). The animal protocol was approved by the Ethics Committee of Shanghai Ninth People's Hospital.

### Materials and Reagents

Medical Mg rods were purchased from the Zhuo Cha new material Technology Co., Ltd. (Xian, China). The rods were wire‐drawn to obtain Mg wires with diameters of 1 and 0.5 mm. The Mg wires were then polished using 2000 grit sandpaper, and ultrasonically washed with acetone, absolute ethanol, and deionized water for 15 min, followed by drying in an oven at 100 °C. Durazane 1500SC was purchased from Honghai Chemical Technology Co., Ltd. (Guangzhou, China). CaCO_3_ nanoparticles (40–80 nm) were obtained from XFNANO Technology Co., Ltd (Nanjing, China). Acetone, butyl acetate, and absolute ethanol were obtained from Sinopharm Chemical Reagent Co., Ltd. De‐ionized water (18.3 MΩ cm^−1^) was prepared using a Milli‐Q purification system (St. Louis, MO, USA) and used in all experiments.

RPMI 1640 medium, Dulbecco's modified Eagle's medium (DMEM), and fetal bovine serum (FBS) were obtained from Gibco Life Technologies (AG, Switzerland). Trypsin‐EDTA and penicillin‐streptomycin, were purchased from Corning life sciences (Wujiang, China).

### Preparation of Mg‐CaCO_3_ and Mg‐OPSZ Rods

Butyl acetate (6.0 g) and Durazane 1500SC (0.6 g) were mixed well under magnetically stirring in a glass bottle. CaCO_3_ nanoparticles (40–80 nm, 1.4 g) was then added to the glass bottle. CaCO_3_/1500SC coating materials were obtained after magnetically stirring for 30 min at room temperature. Subsequently, the CaCO_3_/1500SC coating materials were dipped onto Mg wires and then cured at 150 °C for 4 h. The as‐prepared Mg wires containing CaCO_3_ were referred to as Mg‐CaCO_3_. Pure organic polysilazane coating samples without CaCO_3_ were prepared using the same method, except for changing the dosage of Durazane 1500SC to 2.0 g. These samples were referred to as Mg‐OPSZ. The as‐prepared Mg‐CaCO_3_ and Mg‐OPSZ wires were then cut to rods with equal length of 4 mm by a surgical forcep.

### Characterization

SEM, EDS, and corresponding EDS‐mapping were recorded on a Hitachi Regulus 8100 electron microscope. XRD pattern was obtained on a Rigaku Ultima IV diffractometer. Hydrogen release was recorded by a hydrogen electrode (MMS‐903995, UNISENSE, DK). The absolute Mg and Ca element was determined on an inductively coupled plasma atomic emission spectrometer (ICP‐MS, Aglient 7800, USA). PH values were recorded by a portable PHSJ‐3F pH meter (INESA Scientific Instrument Co., Ltd, China).

### Measurement of Hydrogen Generation

Qualitative measurement of the hydrogen generation from the Mg, Mg‐CaCO_3_, and Mg‐OPSZ rods in PBS (pH 7.4 and pH 6.8) was conducted using a hydrogen electrode. The hydrogen microelectrode was placed in ultrapure water and polarized overnight with a polarization voltage of 1000 mV. When the voltage drops below 50 mV, the microelectrode was inserted into a certain volume of PBS solution. After the baseline was balanced, Mg, Mg‐CaCO_3_, or Mg‐OPSZ rods were added to the PBS solution and then the hydrogen generation from each sample was recorded every 10 s.

### Cell Culture

4T1 murine breast cancer cells, MC38 murine colon cancer cells, and NIH/3T3 murine embryonic fibroblasts were obtained from Type Culture Collection of Chinese Academy of Sciences (Shanghai, China) and were reported in the previous study.^[^ ^46^ ^]^ CAFs were transformed from NIH/3T3 fibroblasts by supplementing 100 ng mL^−1^ recombinant mouse TGF‐β1 (R&D systems, 7666‐MB) for 48 h.^[^ ^22^ ^]^ Splenocytes were collected from the 8‐week‐old male Balb/c mice and activated with Dynabeads Mouse T‐Activator CD3/CD28 (Thermofisher, 11453D) for 2 days. CD4^+^ T cell were isolated using a CD4^+^ T cell isolation kit (Stemcell, #19 852) and expanded in a medium containing 20 ng mL^−1^ IL‐2 for another 5 days. NIH/3T3 cells were cultured in DMEM medium, 4T1, MC38, and CD4^+^ T cells were cultured in RPMI 1640 medium and all medium were supplemented with 10% FBS and 1% (v/v) penicillin‐streptomycin. Except for additional noted, all cells were culture at 37 °C in a 5% CO_2_ humidified incubator.

### Data Collection

Publicly available RNA‐Seq data of TCGA datasets were downloaded from UCSC xena browser (https://xena.ucsc.edu/).^[^ ^47^ ^]^ Only primary tumor samples were kept. Data on RNA‐seq were transcripts per million (TPM) normalized and log2‐transformed.

### Survival Analysis Associated with CAFs Infiltration

For calculating CAFs infiltration scores in each tumor samples, “ssgsea” was performed on CAFs signature.^[^ ^48^ ^]^ The optimal cut‐off point in the infiltration of CAFs was determined using the R package “survminer”. Then, the determined cut‐off values were used for categorizing sample into CAF‐high and ‐low groups. Prediction of OS was performed by univariate Cox proportional regression analysis. The HR and 95% confidence interval (CI) were calculated.

### DEGs Analysis and Functional Enrichment between CAF‐High and ‐Low Groups

For detecting of DEGs, the edge R package was used.^[^ ^49^ ^]^ Genes with a false detection rate (FDR) < 0.05 and absolute fold change (FC) ≥ 2 were considered as differentially expressed. Functional annotation of DEGs was performed on Gene Ontology (GO) and KEGG classification databases by the R clusterProfiler (v3.14.3) package. GO or KEGG categories with a FDR of <0.05 were significantly enriched.

### In Vitro H_2_ Gas Treatment Model^[^ ^25^ ^]^

Mg rods were laid into a 6‐well microplate filled with 1 mL DMEM medium in each well. Plate inserts fitting the 6‐well microplates were coated with polycarbonate membrane and seeded with CAFs. The Mg rods and medium in the well did not directly contact to the upper inserts, for excluding other interrupting factors. The control group was set without any supplement or Mg rods. After incubation for 24 h, cells in the upper inserts were collected for further analysis.

### RNA Sequencing Analysis

Whole RNA was extracted from the indicated cell samples with an RNeasy Plus Mini Kit (Qiagen) according to the manufacturer's protocol. RNA was subjected to RNA‐Seq analysis on a BGISEQ‐500 system by Beijing Genomics Institute (BGI), China. In addition, the RNA was sheared and reverse‐transcribed through random primers to obtain cDNA for library construction. Subsequently, sequencing was performed on the prepared library.^[^ ^50^ ^]^ All the generated raw sequencing reads were filtered to obtain clean reads stored in FASTQ format.^[^ ^51^ ^]^ Bowtie2 and HISAT were used to map clean reads to reference genes and genomes, respectively.^[^ ^52^ ^]^ RSEM was used to quantify the gene expression level (FPKM).^[^ ^53^ ^]^ The NOISeq method was used to screen out DEGs between two groups with a fold change ≥2 and divergence probability ≥0.8. GO and pathway annotation and enrichment analyses were based on the Gene Ontology Database (http://www.geneontology.org/) and KEGG pathway database (http://www.genome.jp/kegg/), respectively.

### *Nos2* Regulation Assay

*Nos2* expression was inhibited by siRNA targeting *Nos2* gene (Genepharma). SiRNA transfection was conducted on CAFs by using the siRNA (100 nm) and Lipo3000 (Thermofisher, L3000075, 2 µL mL^−1^) according to manufacturer's instructions. *Nos2* expression was elevated by adding the NO donor DETA/NO (MCE, HY‐136278) at various concentration.^[^ ^26^ ^]^

### In Vitro H_2_ Gas Therapy by Mg‐CaCO_3_

As described above, Mg‐OPSZ and Mg‐CaCO_3_ rods were laid into a 6‐well microplate filled with 1 mL DMEM medium in each well. 300 µm Mg^2+^ was added in Mg group culture medium, 1 mM CaCO_3_ was added in CaCO_3_ group culture medium. Plate inserts fitting the 6‐well microplates were coated with polycarbonate membrane and seeded with 4T1 cells or CAFs. The medium in the well contacted with the upper inserts. After incubation for 24 h, cells in the upper inserts were collected for further analysis. CCK8 assay was conducted to determine cell viability. Cells with different treatments were then seeded in 96‐well plates (1 × 10^4^ cell per well). If not additionally noted, cells were incubated for 24 h and followed with CCK8 assay. For evaluation of cell viability with different Mg^2+^ concentration, CaCO_3_ concentration, and pH levels, cells were divided into groups and treated with culture medium with different ion concentrations and pH levels as indicated. The apoptosis assay was performed using flow cytometry (BD LSRFortessa) after Annexin V/PI staining (556 547, BD Biosciences) following manufacturer's instructions. For detection of CRT, treated cells were stained with anti‐Calreticulin antibody (Abcam, ab92516, 1:100) and Goat Anti‐Rabbit IgG H&L (Alexa Fluor 488) (Abcam, ab150077, 1:1000) for 30 min, and further analyzed by flow cytometry.

For the measurement of intracellular hydrogen release, treated cells continued to co‐incubate with 40 µm of MB solution for 12 h. After incubation, the medium was removed, and the cells were gently washed three times with PBS. Then images were obtained by a microscope. The change of MB concentration was recorded by the absorption intensity in UV–vis spectra.^[^ ^11^ ^]^ For the measurement of intracellular pH level, treated cells were stained with BCECF AM probe (Beyotime, #S1006, 5 µm) for 30 min, and were further analyzed by flow cytometry. For intracellular ROS detection, treated cells were stained with DCFH‐DA probe (Beyotime, #S0033, 10 µm) for 30 min, and were further analyzed by flow cytometry. For the detection of mitochondria membrane potential, treated cells were stained with JC‐1 (monomer: green, aggregation: red) (Beyotime, C2003S) and fluorescent images were acquired by Confocal Laser Scanning Microscope (CLSM, Leica TCS SP2; Leica Microsystems, Heidelberg, Germany).

### CAFs’ Phenotypes Analysis

For real‐time qPCR analysis, RNA was extracted from cell lines by using RNA isolation kit according to the manufacturer's instructions (Yeasen, 19221ES). Reverse transcription was performed using the PrimeScriptTM RT Reagent Kit with gDNA Eraser (TaKaRa #RR047Q). Real‐time qPCR was conducted using SYBR Green qPCR Master Mix (Bimake, B21702) and a QuantStudio 7 Flex Real‐Time PCR System (Thermo Fisher). The primer sequences can be found in Table S1 (Supporting Information).

For western blotting assays, proteins were extracted from cultured cells using cold RIPA lysis buffer (Thermo Fisher, 89 901) and protease inhibitor cocktail (Bimake, B14001) and a phosphatase inhibitor cocktail (Bimake, B15001). Proteins from cell lysates were separated by SDS‐PAGE and then transferred to PVDF membranes (Bio‐Rad). The blots were incubated with primary antibodies: anti‐FN1 (Proteintech, 15613‐1‐AP, 1:2000), anti‐COL1A1 (CST, #72 026, 1:1000), anti‐VIM (Proteintech, 10366‐1‐AP, 1:2000), anti‐NOS2 (Proteintech, 80517‐1‐RR, 1:2000), anti‐IRF1 (CST, #8478, 1:1000), and anti‐β‐Actin (CST, #3700, 1:1000) at 4 °C overnight and with secondary antibodies at RT for 1 h. Blot images were captured by ODYSSEY imaging system.

For immunocytochemistry assay, treated CAFs were fixed with 4% paraformaldehyde, immersed into 0.3% hydrogen peroxide, blocked with 10% goat serum, and incubated with a specific primary antibody at 4 °C overnight: anti‐FN1 (Proteintech, 15613‐1‐AP, 1:200), anti‐COL1A1 (CST, #72026, 1:200), and anti‐α‐SMA (Abcam, Ab7817, 1:1000). Then the cells were incubated with secondary antibodies at RT for 1 h. Fluorescent images were acquired by Confocal Laser Scanning Microscope (CLSM, Leica TCS SP2; Leica Microsystems, Heidelberg, Germany).

ELISA was used to measure immune‐related factors produced by CAFs. The cell culture supernatant was collected from the plate and mice Cxcl1 and Cxcl12 were measured by ELISA kits according to the manufacturer's protocol.

### CD4^+^ T Cell Co‐Culture Model and Analysis

CD4^+^ T cells were seeded in a 6‐well microplate (1.5 × 10^5^ cell per well). Plate inserts with treated CAFs were put into wells. After incubation for 24 h, CD4^+^ T cells in the wells were collected for further PCR and flow cytometry analysis. The antibodies used for flow cytometry were ICOS (Bio Legend, #313 505, 1:100) and Foxp3 (Bio Legend, #126 407, 1:100). The primer sequences can be found in Table S1 (Supporting Information).

### Animals

Balb/c nude, Balb/c,s and C57BL/6 mice were purchased from Shanghai Jihui Laboratory Animal Care Co. Ltd. All the experimental procedures and plans were carefully reviewed and approved by the Animal Ethical Committee of Shanghai Ninth People's Hospital. All the model mice were raised in a standard environmentally controlled room (23 °C, with 55 ± 5% humidity and under a 12 h/12 h light/dark cycle).

### Synergic Tumor Implantation Model

For the anti‐tumor effect study, 100 µL of PBS containing 2 × 10^6^ 4T1 cells were subcutaneously injected into the right flanks of mice. When the tumor volumes of the mice reached ≈150 mm^3^, the mice were randomly divided into three groups (n = 5 per group) and intratumorally implanted with different samples: a) Ctrl group, b) Mg‐OPSZ, and c) Mg‐CaCO_3_. The Ctrl group was set as blank control. The tumor volumes were recorded every two days. The tumor volume was measured via the following equation: V = (width)^2^ × length/2. Then the mice was sacrificed and the tumors were collected for photographing and weighing.

### Bilateral Tumor Implantation Model

For the systematic immune effect study, an orhotopic tumor metastasis model was established by subcutaneous injecting 1× 10^6^ 4T1 cells or MC38 cells per mouse into the right flank and 5 × 10^5^ 4T1 cells or MC38 cells per mouse into the left flank of mice. When the primary tumor (on the right flank) reached ≈150 mm^3^, the mice were randomly divided into three groups (n = 5 per group) and intratumorally implanted with different samples in the primary tumors: a) Ctrl group, b) Mg‐OPSZ, and c) Mg‐CaCO_3_. The Ctrl group was set as blank control. The tumor volume of both sides were measured every two days. After two weeks, the mice were sacrificed, and the subsequent determinations were carried out as the procedure mentioned above.

### Combination Treatment Model

For the combination treatment model, 1 × 10^6^ 4T1 cells were subcutaneously injected into the right flanks of mice. When the tumor volumes of the mice reached ≈100 mm^3^, the mice were randomly divided into three groups (n = 6 per group): Ctrl, αPD1, and Mg‐CaCO_3_+αPD1. Mice in the Mg‐CaCO_3_+αPD1 group were intratumorally implanted with Mg‐CaCO_3_ in the day 0. Mice in the αPD1 and Mg‐CaCO_3_+αPD1 groups were intraperitoneally injected with αPD1 antibody (10 mg kg^−1^) in the day 1, 3, and 5 while mice in the Ctrl group were injected with 200 µL PBS. The tumor volumes were recorded every three days. Mice were monitored for 60 days and the survival rates were expressed using Kaplan‐Meier plots.

### Ultrasonic Imaging

For in vivo ultrasonic imaging, 4T1 tumor‐bearing mice were imaged under the Vevo LAZR small animal ultrasonic imaging system after implantation with one Mg‐OPSZ or Mg‐CaCO_3_ rod.

### Analysis of Histopathology, IHC, and IF Staining

For H&E staining, the hearts, livers, spleens, lungs, kidneys, and tumors were fixed in 10% formalin and then embedded in paraffin. Afterwards, the tissues embedded in paraffin were cut into slices and stained with H&E for observation under an optical microscope. For IHC analysis, tumor sections were formalin‐fixed and stained with primary anti‐ki67 (Servicebio, GB111141, 1:100), anti‐CD4 (Abcam, ab183685, 1:1000), anti‐CD8 (Abcam, ab217344, 1:2000) antibodies, via a typical IHC procedure. For TUNEL assay, the paraffin slices of tumor tissues were de‐waxed using xylene, hydrated in graded ethanol, and then the slices were incubated with proteinase K at 37 °C for 20 min, the obtained slices were rinsed using PBS (pH 7.4) and stained with TUNEL at 37 °C for 1 h in the dark. Subsequently, Dapi was used to stain the nuclei of tumor cells. Then the tumor cell apoptosis in slices of various groups was observed on a CLSM. For IF assay, tumor sections were stained with primary anti‐COL1A1 (CST, #72 026, 1:200), anti‐CD4 (Abcam, ab183685, 1:200), and anti‐NOS2 (Proteintech, 80517‐1‐RR, 1:200) antibodies and then second antibodies. DCFH‐DA probe was used to evaluate ROS generation and Thiol Tracker violet to evaluate the GSH content in each group.

### In Vivo Evaluation of Immunological Response

The primary tumors and distant tumors were harvested in the end of experiments to analyze the CD4^+^ and CD8^+^ T cells infiltration in tumors for the bilateral tumor‐bearing mice. For flow cytometry analysis, the harvested tumors were shredded and digested. Erythrocyte lysate was added to remove red cells. The single‐cell suspension was obtained after filtration by nylon mesh filters. To analyze the T cells in tumors, the cells were stained with anti‐CD45‐FITC (Bio Legend, #103 107, 1:100), anti‐CD4‐PEcy7 (Bio Legend, #100 422, 1:100), anti‐CD8a‐PE (Bio Legend, #162 304, 1:100), anti‐PD1‐percpcy5.5 (Bio Legend, #135 207, 1:100), anti‐CTLA4‐BV605 (Bio Legend, #106 323, 1:100), and anti‐Foxp3‐AF647 (Bio Legend, #126 407, 1:100). These samples were observed on a flow cytometry and obtained data were analyzed using Flowjo software. The collected blood was centrifuged under low temperature to obtain the serum. Mice ifn‐γ and tnf‐α in serum was measured by ELISA kits according to the manufacturer's protocol.

### Biodegradability and Biocompatibility Study

4T1 tumor‐bearing mice were implanted with Mg‐OPSZ and Mg‐CaCO_3_ rods intratumorally (n = 3). Body weights of mice were measured every 2 days. Tumors and major organs including heart, liver, spleen, lung, and kidney of each mice were dissected at different time points. Organs tissues were fixed in 4% neutral buffered formalin, embedded into paraffin, sectioned by routine procedures for further H&E staining. Tumors and organ tissues were weighed, solubilized in aqua regia and measured by ICP‐MS to determine Ca and Mg levels in them. The blood was also collected and weighed, solubilized in aqua regia, and measured by ICP‐MS to determine Ca and Mg levels. Meanwhile, the blood samples were collected for blood biochemistry tests, which were conducted by Servicebio Biotechnology Co., Ltd.

### Statistical Analysis

All data were presented as mean ± SD. Statistical analysis was performed with GraphPad Prism 8.0.1 software by two‐tailed unpaired Student's *t*‐tests, log‐rank test, or one‐way ANOVA.

## Conflict of Interest

The authors declare no conflict of interest.

## Supporting information

Supporting Information

## Data Availability

The data that support the findings of this study are available from the corresponding author upon reasonable request.
