# Supplementary material for: Hydrogen Therapy Reverses Cancer‐Associated Fibroblasts Phenotypes and Remodels Stromal Microenvironment to Stimulate Systematic Anti‐Tumor Immunity
Source: Adv Sci (Weinh). 2024 May 17;11(28):2401269. doi: 10.1002/advs.202401269 (PMC11267370; doi:10.1002/advs.202401269)
Supplement: Supplementary file 1 — Supporting Information [file ADVS-11-2401269-s001.docx]

Supporting Information

**Hydrogen therapy reverses cancer-associated fibroblasts phenotypes and remodels stromal microenvironment to stimulate systematic anti-tumor immunity**

Xiaoyan Meng, Zhonglong Liu, Liang Deng, Yangzi Yang, Yingchun Zhu, Xiaoying Sun*, Yongqiang Hao*, Yue He*, and Jingke Fu*

X. Y. Meng, Dr. Z. L. Liu, Prof. Y. He

Department of Oral Maxillofacial & Head and Neck Oncology, Shanghai Ninth People’s Hospital, Shanghai Jiao Tong University School of Medicine; College of Stomatology, Shanghai Jiao Tong University; National Center for Stomatology; National Clinical Research Center for Oral Diseases; Shanghai Key Laboratory of Stomatology, Shanghai 200011, P.R. China;

E-mail: william5218@126.com

Dr. L. Deng, Dr. J. K. Fu, Prof. Y. Q. Hao

Shanghai Key Laboratory of Orthopaedic Implant, Department of Orthopaedic Surgery, Shanghai Ninth People’s Hospital, Shanghai Jiao Tong University School of Medicine; Clinical and Translational Research Center for 3D Printing Technology; Shanghai Engineering Research Center of Innovative Orthopaedic Instruments and Personalized Medicine, Shanghai, 200011, China;

E-mail: [fujingke@sjtu.edu.cn](mailto:fujingke@sjtu.edu.cn); hao_yongqiang@hotmail.com.

Y. Z. Yang

Department of Orthopedic Surgery, Spine Center, Changzheng Hospital, Navy Medical University, No. 415 Fengyang Road, Shanghai 200003, China;

Prof. Y. C. Zhu

Key Laboratory of Inorganic Coating Materials, Shanghai Institute of Ceramics, Chinese Academy of Sciences, 200050 Shanghai, China;

Dr. X. Y. Sun

College of Sciences, Shanghai University, Shanghai 200444, China.

E-mail: xysun@shu.edu.cn.

This file includes:

Supplementary Figure 1-30

Supplementary Table 1


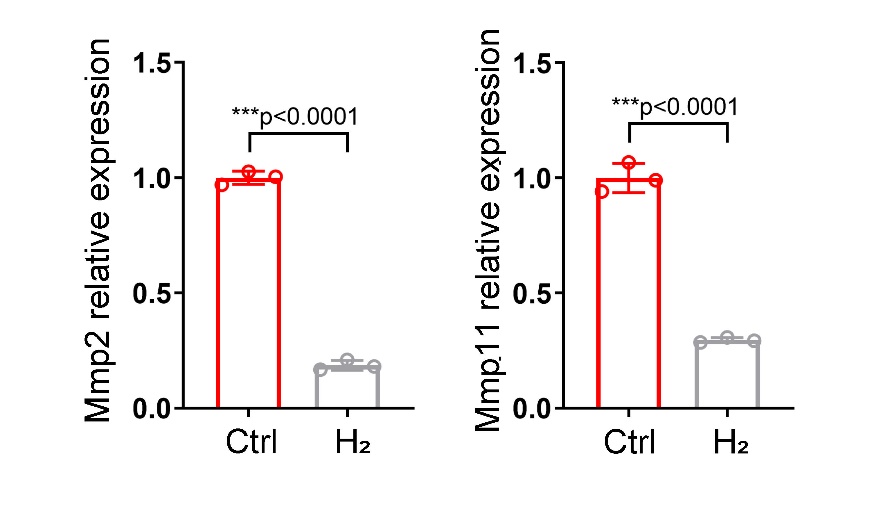


**Figure S1** MRNA levels of pro-tumor factors analyzed by qPCR in CAFs with (H_2_) or without (Ctrl) hydrogen treatment (n = 3). Statistical significance was calculated *via* two-tailed Students’ *t*-test. ****p* < 0.001. The mean values and SD are presented.


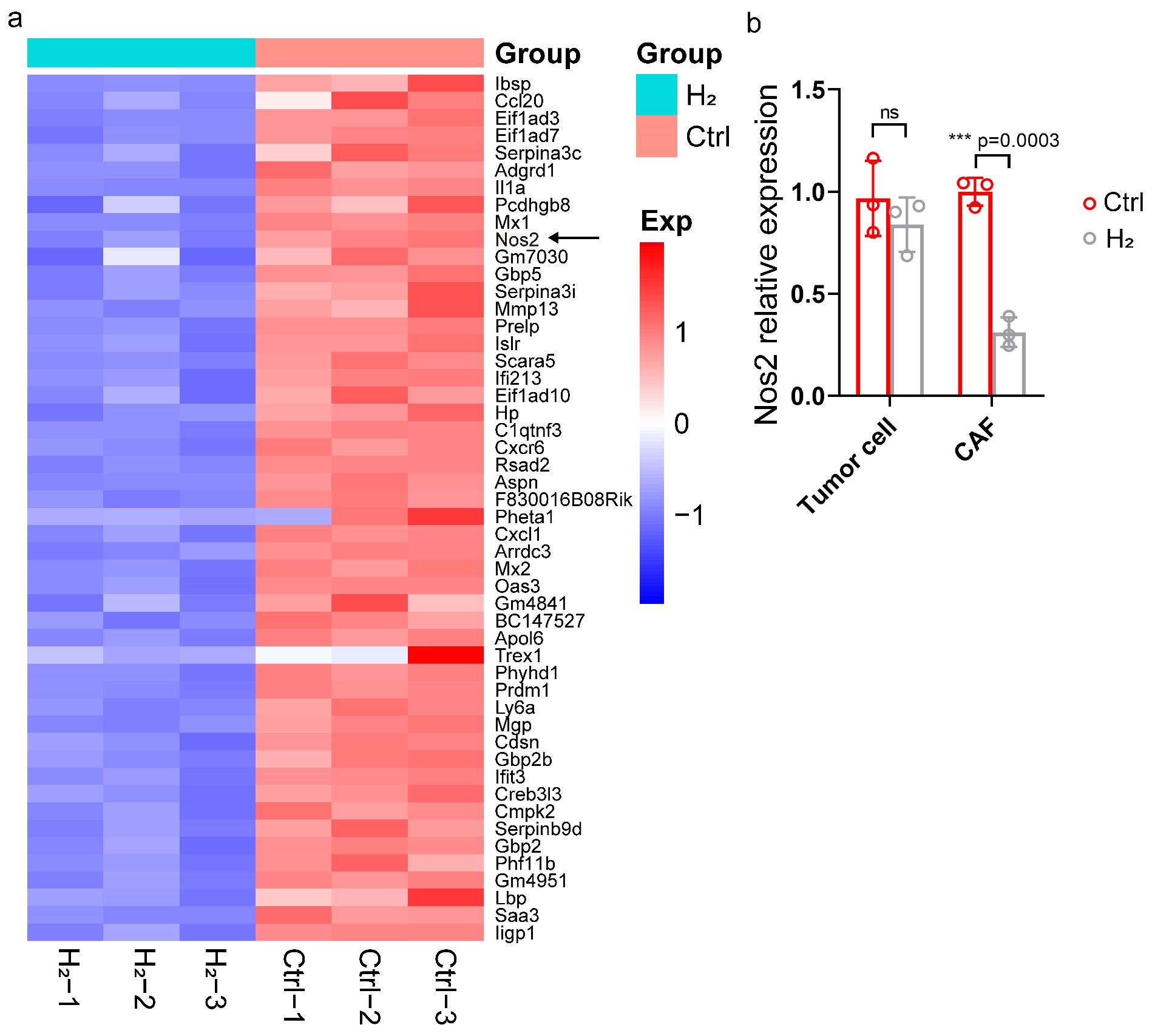


**Figure S2** (a) Heatmap shows top 50 down-regulated DEGs in CAFs with hydrogen treatment (H_2_) compared to those without hydrogen treatment (Ctrl) (n = 3) (*Nos2* was indicated by the arrow). (b) MRNA level of *Nos2* analyzed by qPCR in tumor cells and CAFs with (H_2_) or without (Ctrl) hydrogen treatment (n = 3). Statistical significance was calculated *via* two-tailed Students’ *t*-test. ****p* < 0.001. The mean values and SD are presented.


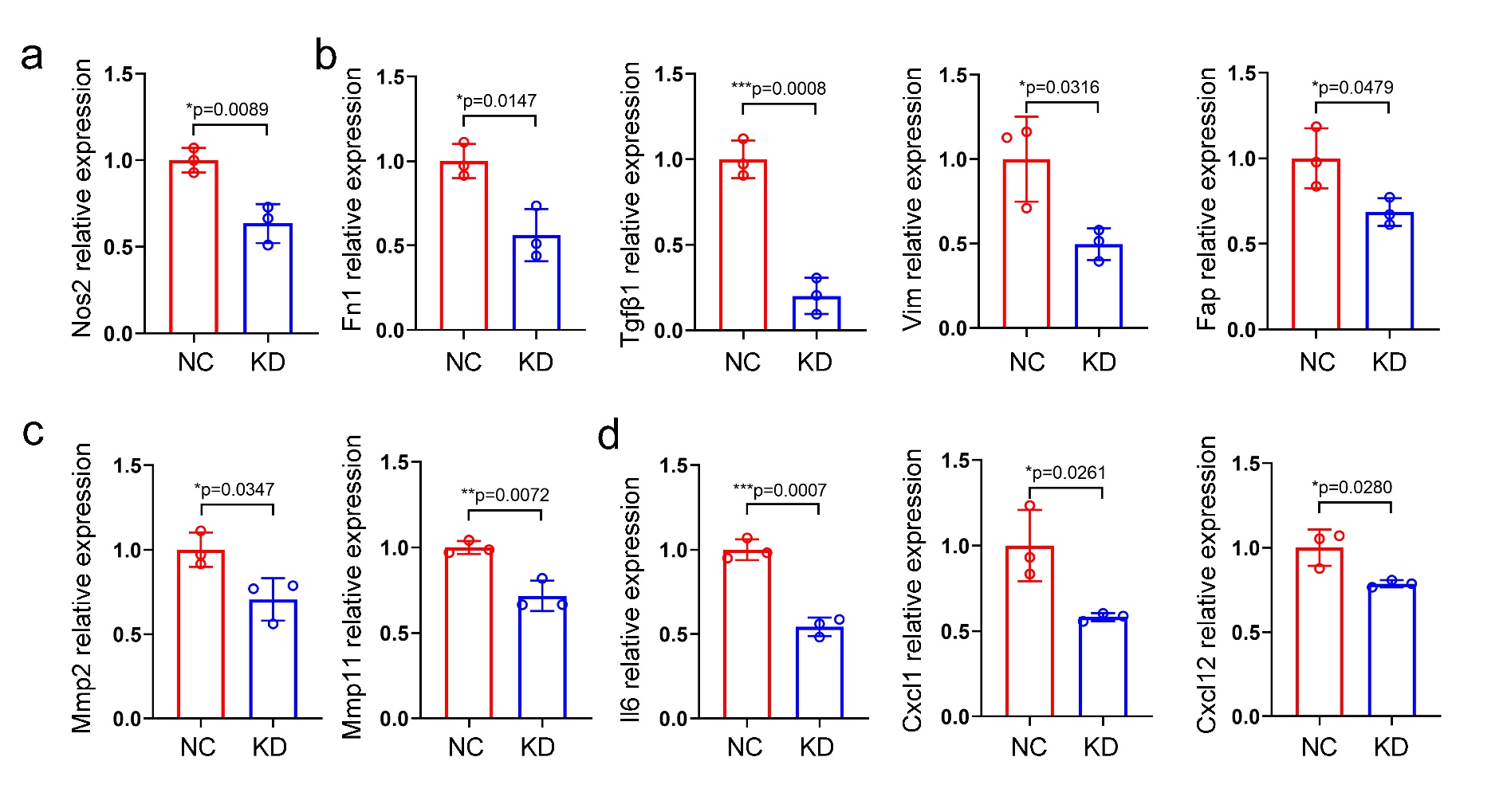


**Figure S3** MRNA levels of *Nos2* (a), CAF markers (b), pro-tumor factors (c), and immune-modulatory factors (d) analyzed by qPCR in the CAFs with (KD) or without (NC) *Nos2* knockdown (n = 3). Statistical significance was calculated *via* two-tailed Students’ *t*-test. **p* < 0.05, ***p* < 0.01, and ****p* < 0.001. The mean values and SD are presented.


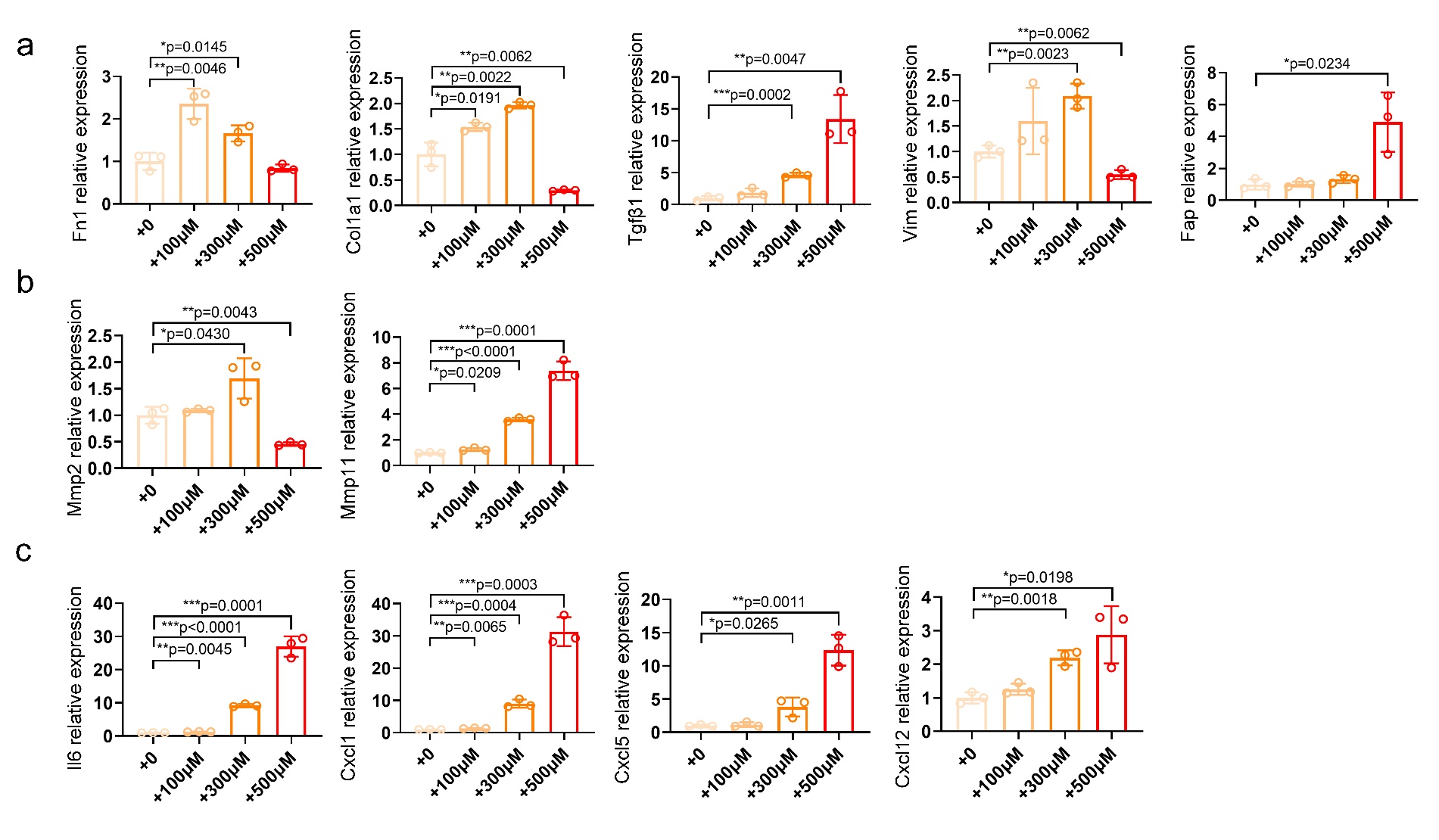


**Figure S4** MRNA levels of CAF markers (a), pro-tumor factors (b), and immune-modulatory factors (c) analyzed by qPCR in the CAFs with hydrogen treatment then DETA/NO treatment in different concentrations (0, 100 μM, 300μM, and 500 μM) (n = 3). Statistical significance was calculated *via* two-tailed Students’ *t*-test. **p* < 0.05, ***p* < 0.01, and ****p* < 0.001. The mean values and SD are presented.





**Figure S5** SEM image of the CaCO_3_ nanoparticles. Scale bar = 200 nm.


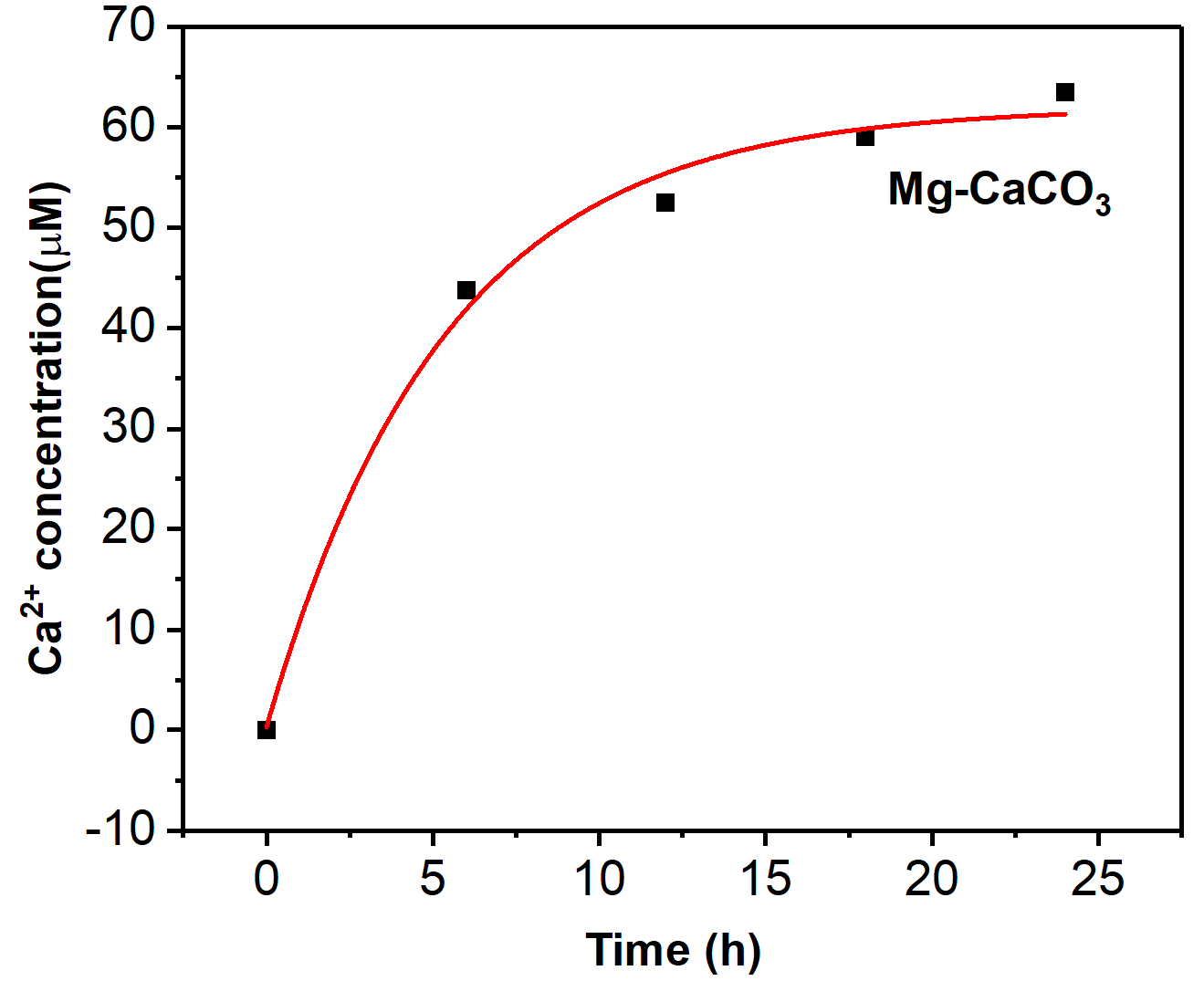


**Figure S6** Ca ions release profiles from Mg-CaCO_3_ rods in PBS solution measured by ICP-MS.


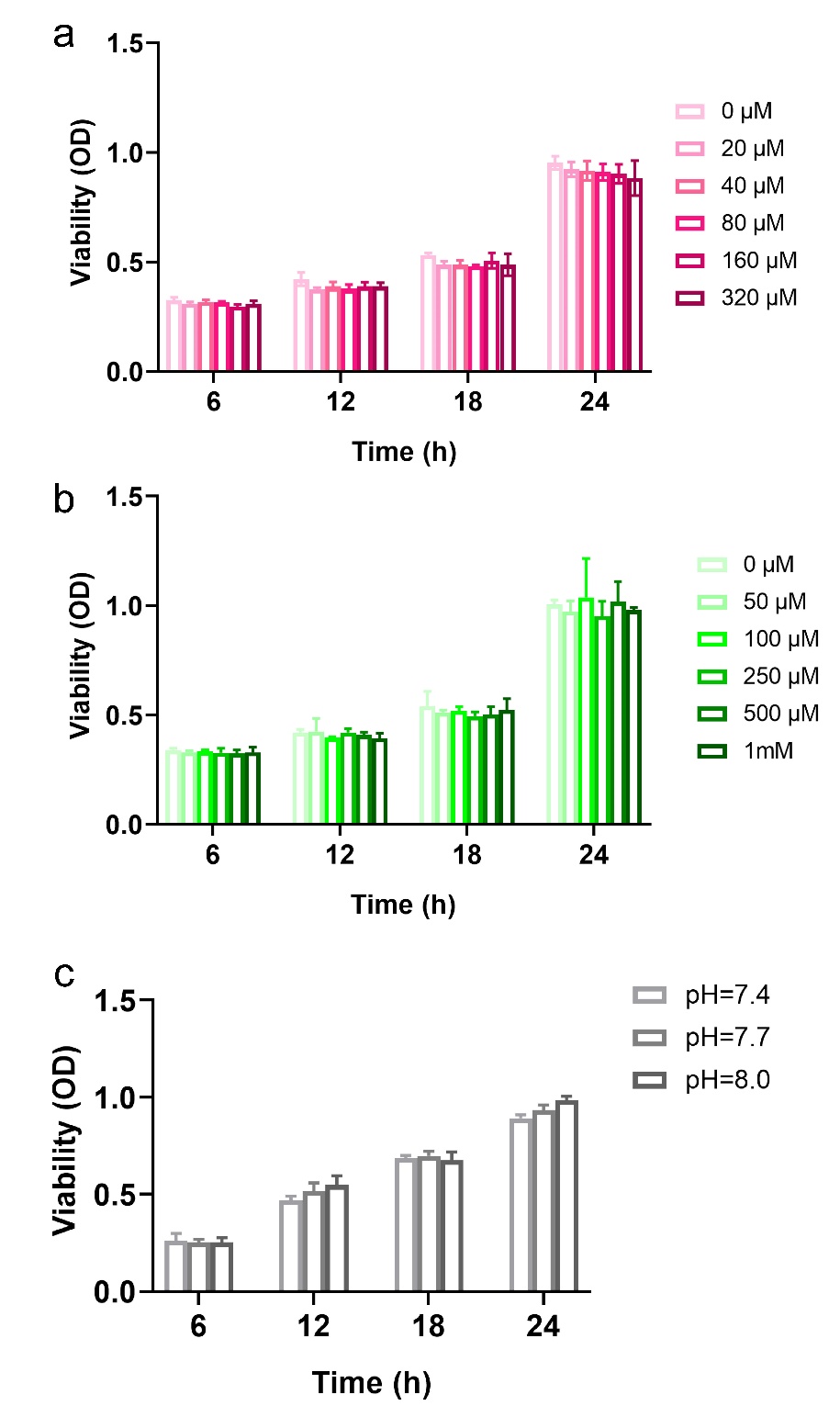


**Figure S7** Cell viability of 4T1 cells treated with different Mg^2+^ concentrations (a), CaCO_3_ concentrations, and pH levels (c) at different time points (n = 3). Statistical significance was calculated *via* two-tailed Students’ *t*-test. There is no statistical significance between treatment groups at each time point. The mean values and SD are presented.


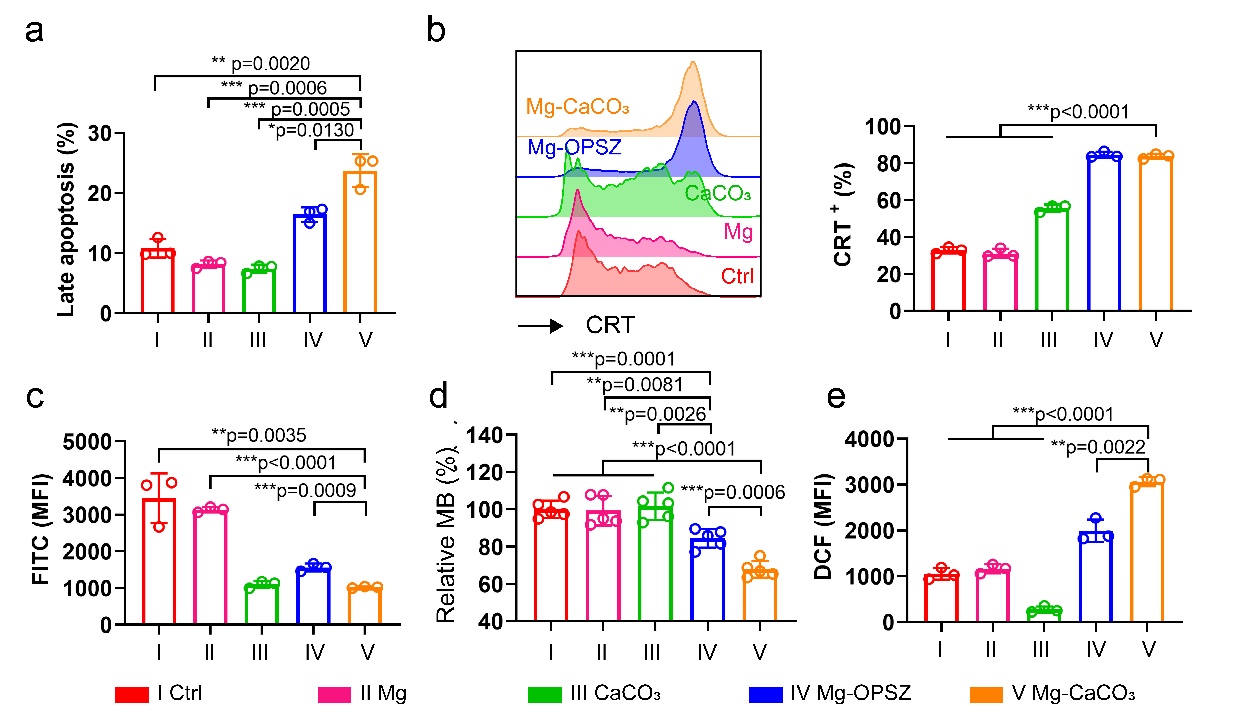


**Figure S8** (a) Quantitative analysis of the extent of 4T1 cells late apoptosis by Annexin V/PI staining (n = 3), related to Figure 3b-c. (b) Flow cytometry assay and corresponding quantitative analysis of the CRT^+^ cell ratio of 4T1 cells (n = 3). (c) Quantitative analysis of mean fluorescent intensity (MFI) of 4T1 cells by BCECF AM probe (n = 3), related to Figure 3d-e. (d) Quantitative analysis of MB reduction within 4T1 cells (n = 5), related to Figure 3f. (e) Quantitative analysis of MFI of 4T1 cells by DCFH-DA probe (n = 3), related to Figure 3h-i. Group: I: Ctrl, II: Mg, III: CaCO_3_, IV: Mg-OPSZ, V: Mg-CaCO_3_. Statistical significance was calculated *via* two-tailed Students’ *t*-test. **p* < 0.05, ***p* < 0.01, and ****p* < 0.001. The mean values and SD are presented.


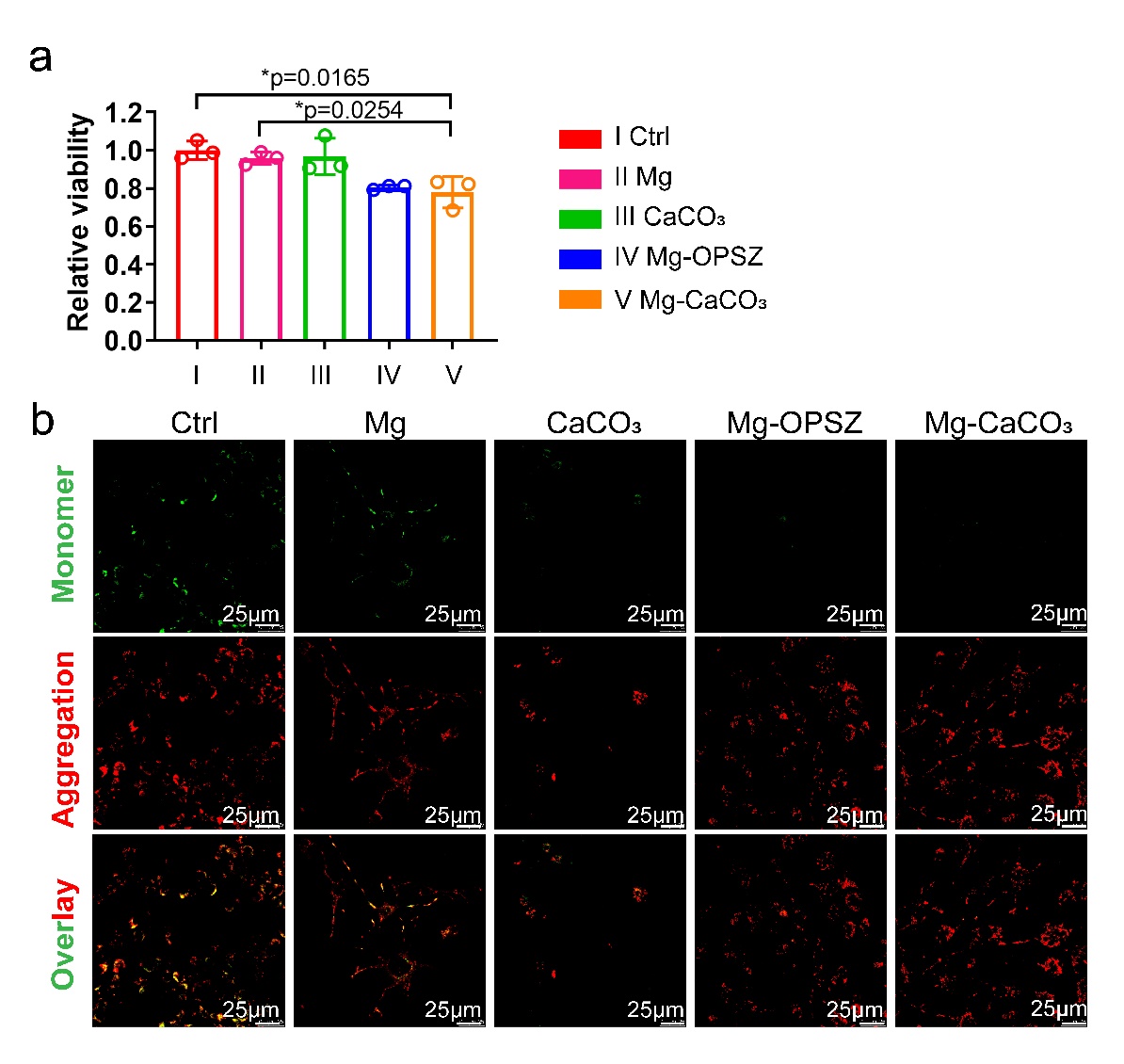


**Figure S9** (a) Relative cell viabilities of CAFs with different treatments for 24 h (n = 3). (b) Fluorescence microscope images of CAFs with different treatments and then stained with JC-1 dye. Scale bar = 25 μm. Group: I: Ctrl, II: Mg, III: CaCO_3_, IV: Mg-OPSZ, V: Mg-CaCO_3_. Statistical significance was calculated *via* two-tailed Students’ *t*-test. **p* < 0.05. The mean values and SD are presented.


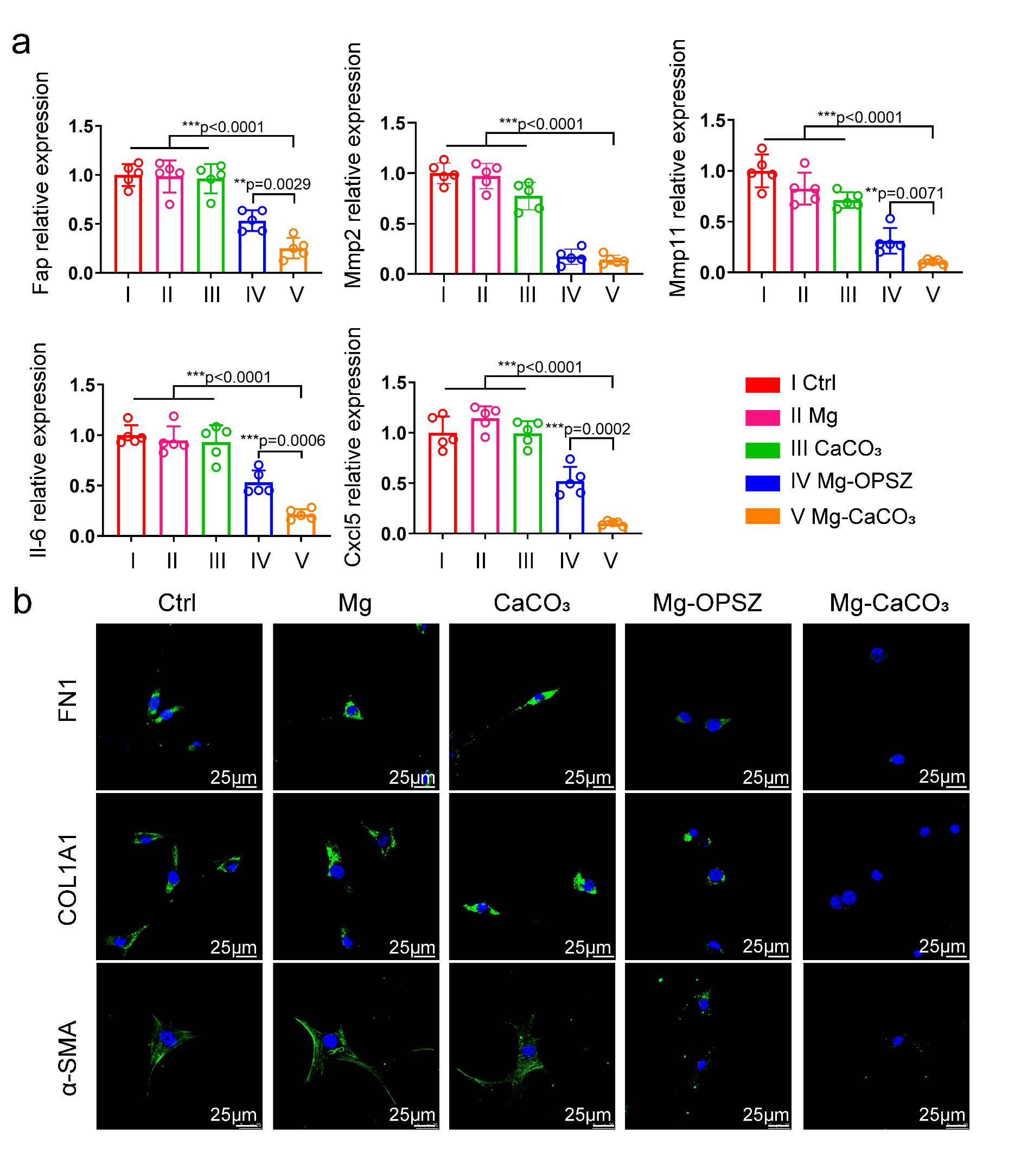


**Figure S10** (a) MRNA level of different genes analyzed by qPCR in CAFs with different treatments (n = 5). (b) Fluorescence microscope images of CAFs with different treatments and then stained with antibodies to CAF markers. Green: FN1, COL1A1, and α-SMA, as indicated, blue: DAPI, Scale bar = 25 μm. Group: I: Ctrl, II: Mg, III: CaCO_3_, IV: Mg-OPSZ, V: Mg-CaCO_3_. Statistical significance was calculated *via* two-tailed Students’ *t*-test. ***p* < 0.01 and ****p* < 0.001. The mean values and SD are presented.


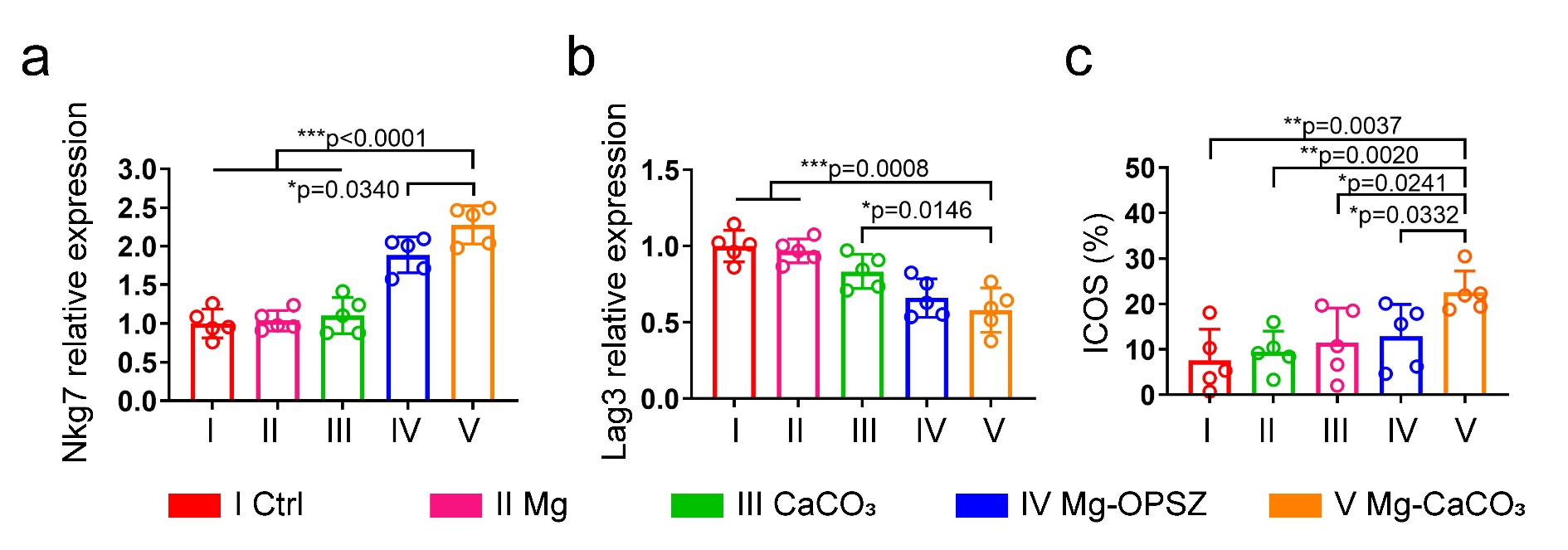


**Figure S11** (a-b) MRNA levels of *Nkg7* (a) and *Lag3* (b) analyzed by qPCR in CD4^+^ T cells co-cultured with CAFs with different treatments (n = 5). (c) Quantitative analysis of ICOS^+^ ratio in CD4^+^ T cells co-cultured with CAFs with different treatments (n = 5). Group: I: Ctrl, II: Mg, III: CaCO_3_, IV: Mg-OPSZ, V: Mg-CaCO_3_. Statistical significance was calculated *via* two-tailed Students’ *t*-test. **p* < 0.05, ***p* < 0.01, and ****p* < 0.001. The mean values and SD are presented.


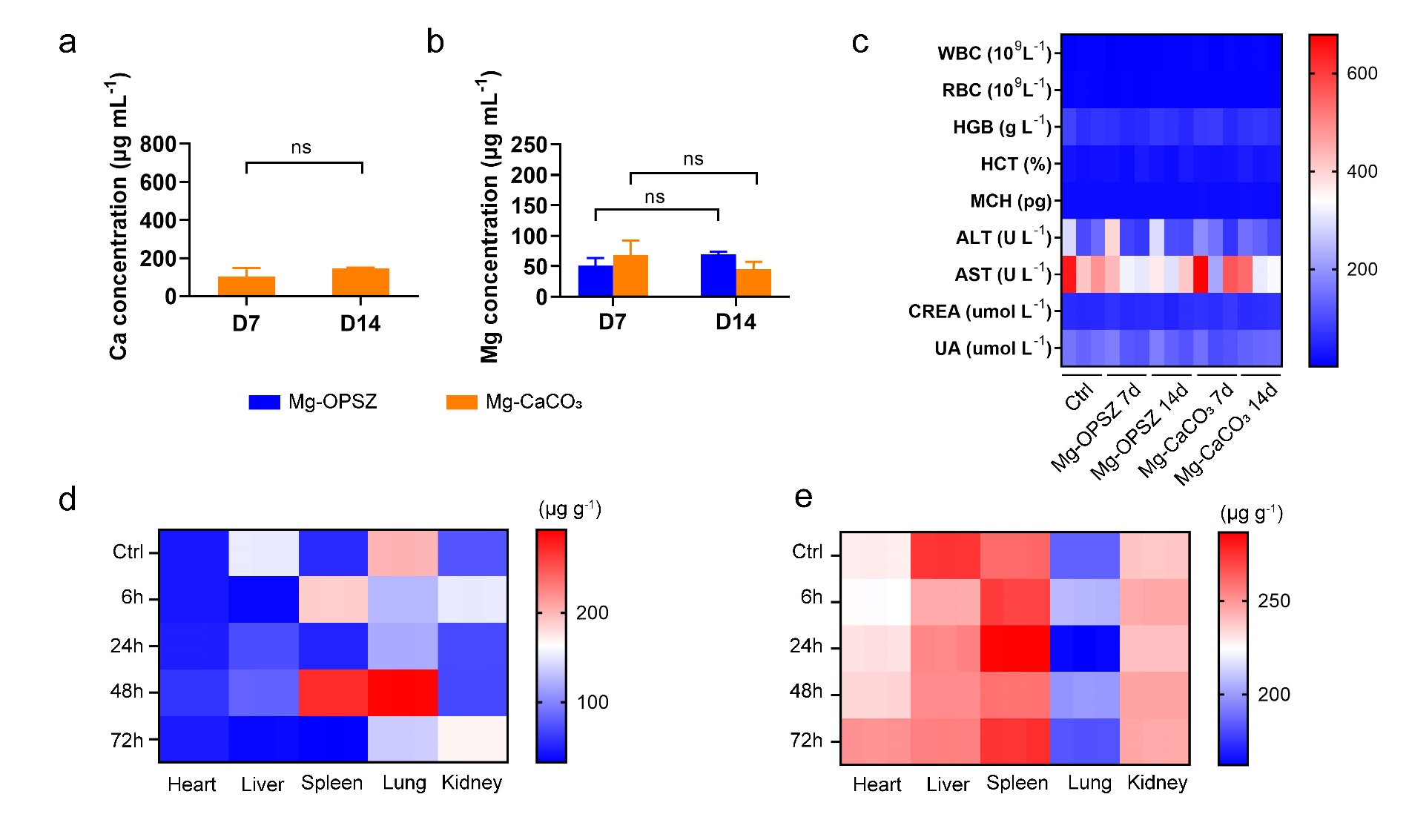


**Figure S12** (a-b) The Ca (a) and Mg (b) levels in peripheral blood of mice after different treatments for 7 days and 14 days (n=3). (c) Heatmap shows blood biosafety parameters of mice after different treatments for 7 days and 14 days. (d-e) Heatmaps show Ca (d) and Mg (e) levels in major organs after Mg-CaCO_3_ treatment at different time points. Statistical significance was calculated *via* two-tailed Students’ *t*-test. The mean values and SD are presented.


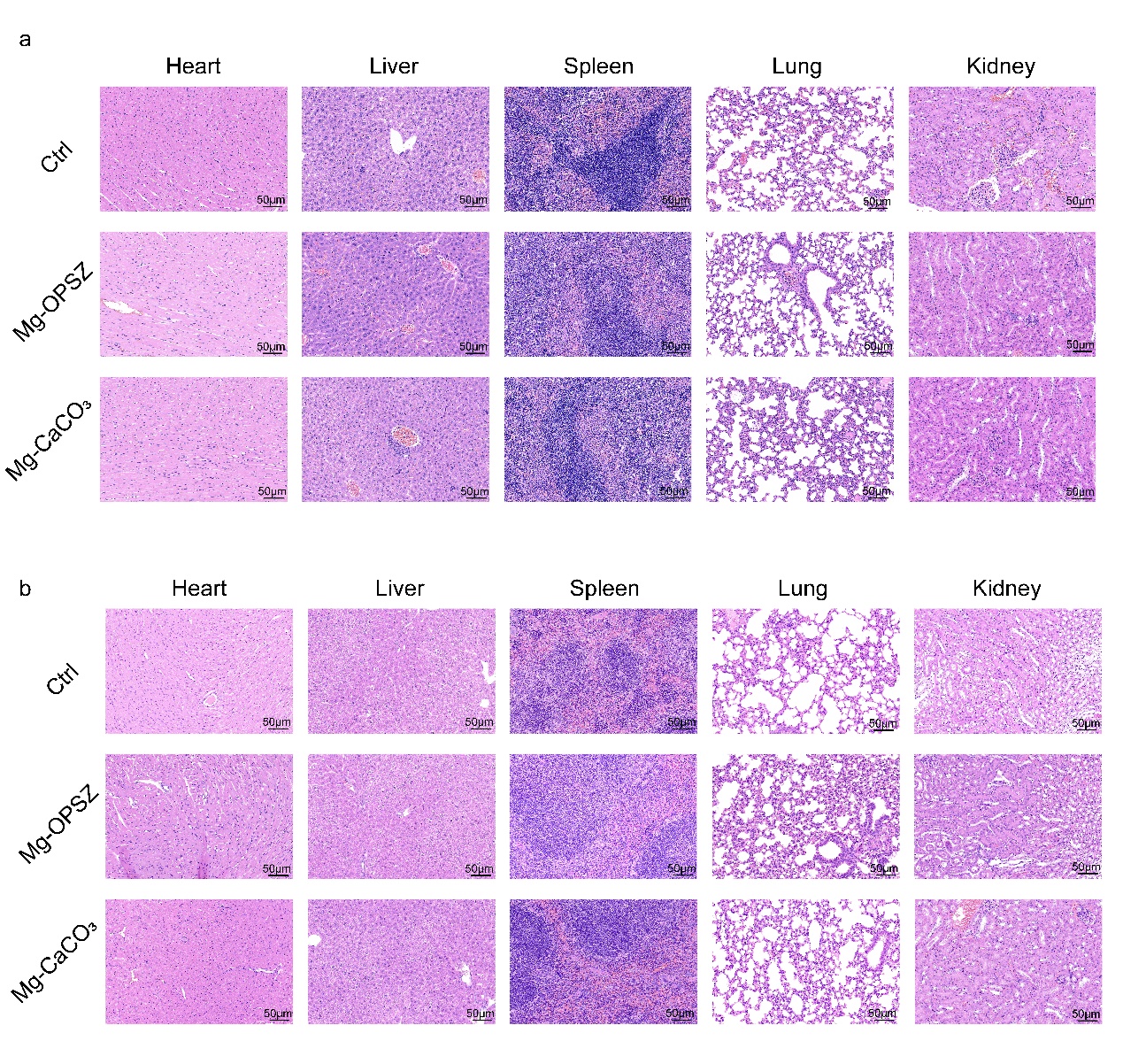


**Figure S13** H&E staining of mouse major organs (heart, liver, spleen, lung, and kidney) to examine the histological changes after different treatments for 7 days (a) and 14 days (b). Scale bar = 50 μm.


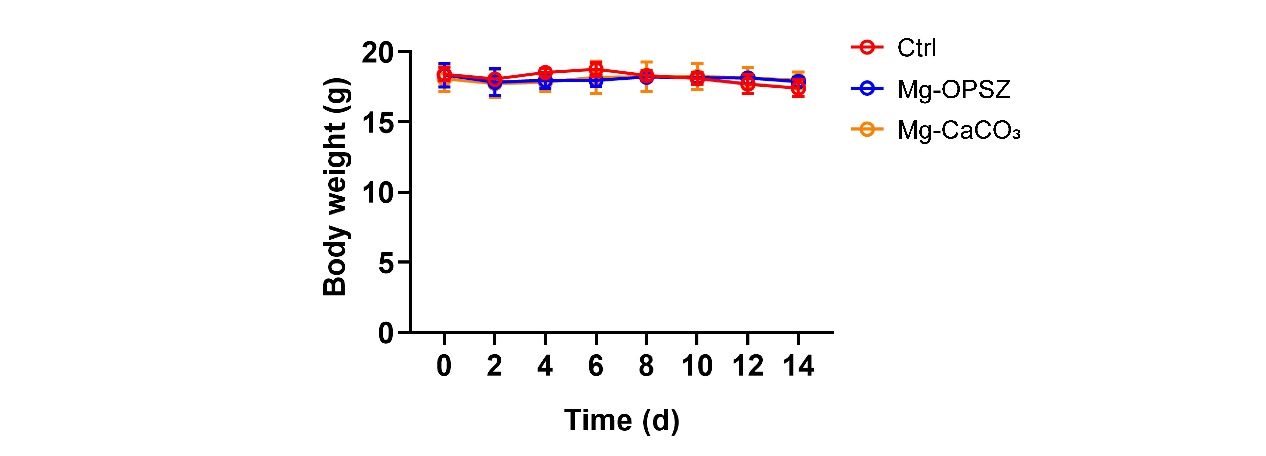


**Figure S14** Body weight fluctuation curves of mice after different treatments for 14 days (n = 3).


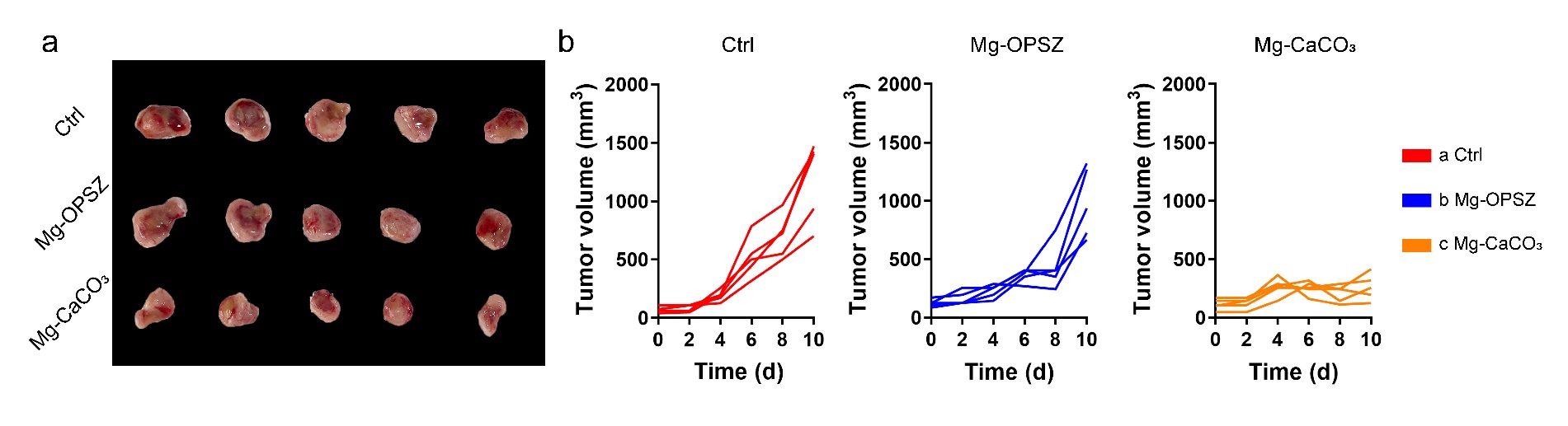


**Figure S15** (a) Representative photographs of tumors collected from Balb/c nude mice after different treatments. (b) Individual tumor growth curves of Balb/c nude mice after different treatments.


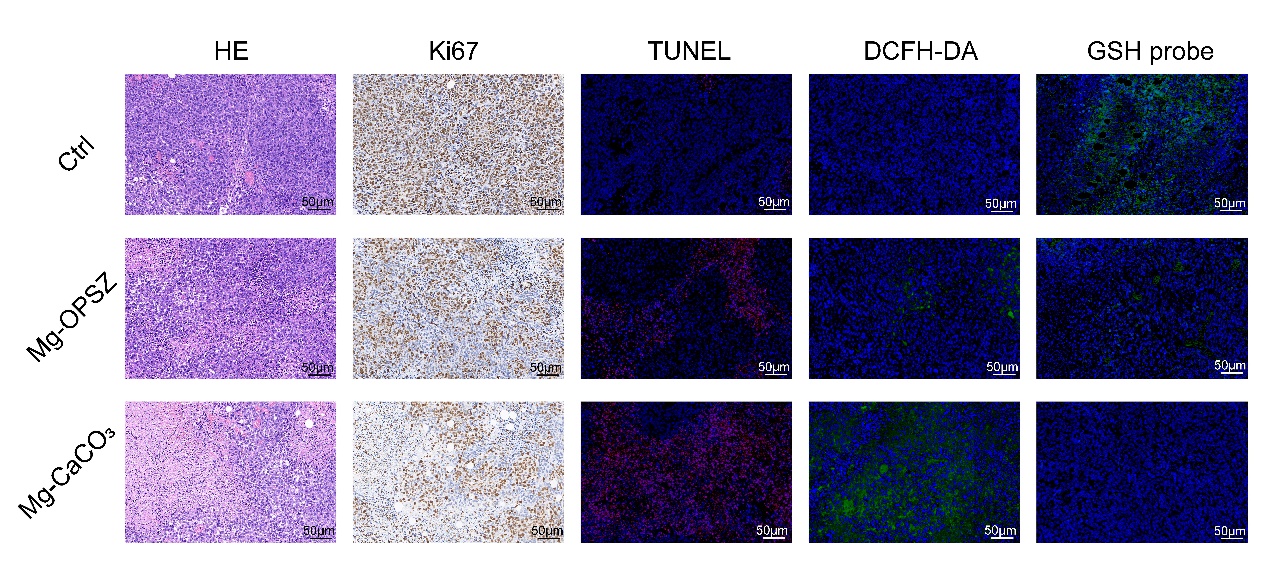


**Figure S16** Microscopy images of H&E, Ki67, TUNEL, DCFH-DA, and GSH probe staining of tumors collected from Balb/c nude mice after different treatments. Scale bar = 50 μm.


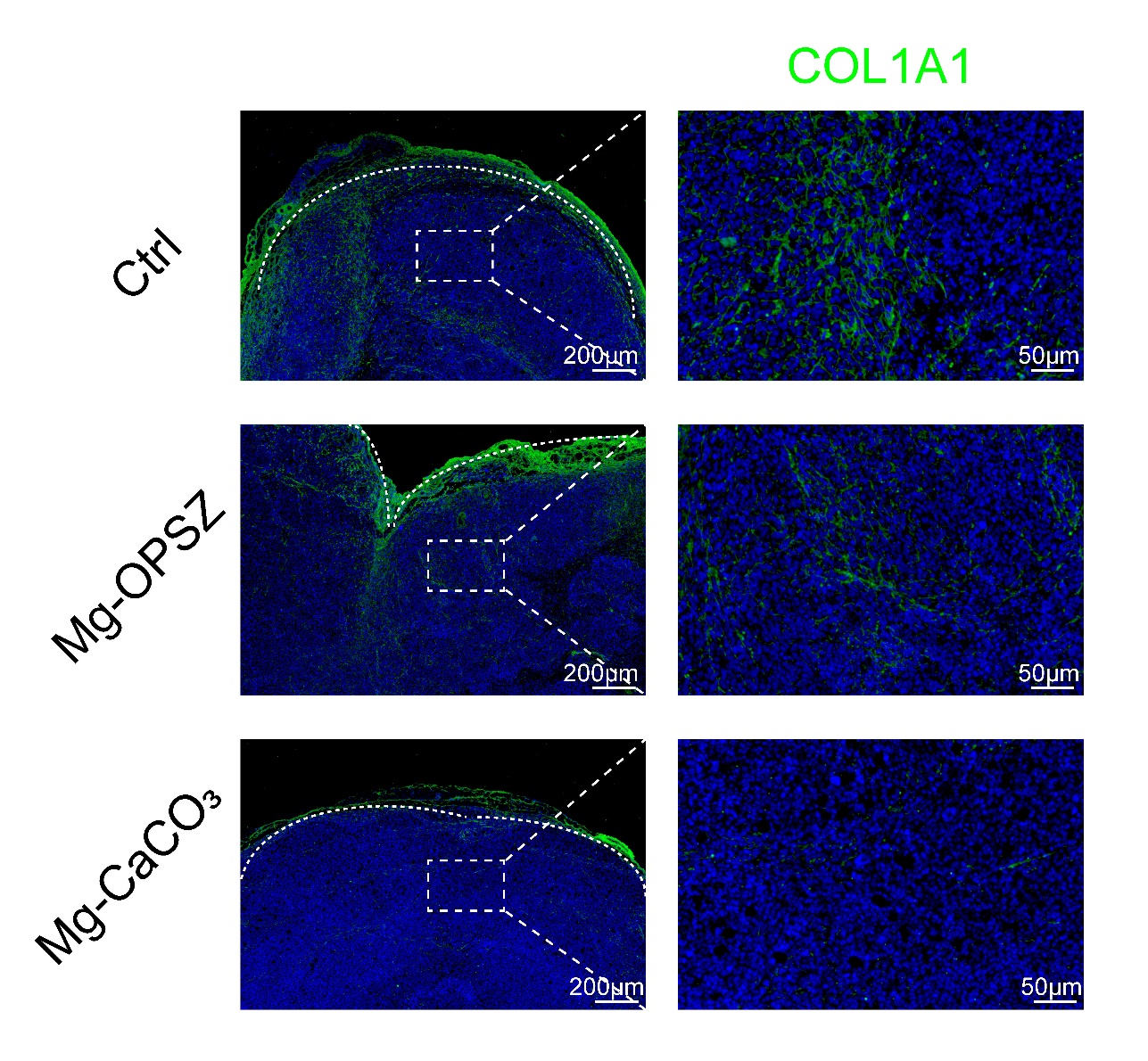


**Figure S17** IF images of tumors collected from Balb/c nude mice after different treatments. Green: COL1A1, blue: DAPI, scale bar: 200 μm and 50 μm, as indicated.


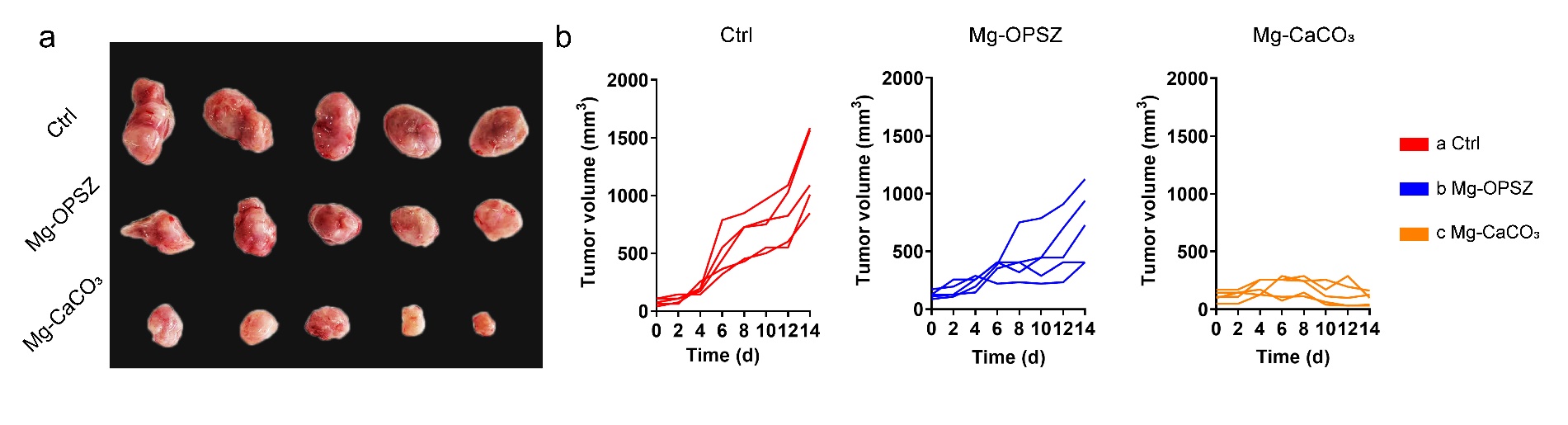


**Figure S18** (a) Representative photographs of tumors collected from Balb/c mice after different treatments. (b) Individual tumor growth curves of Balb/c mice after different treatments.


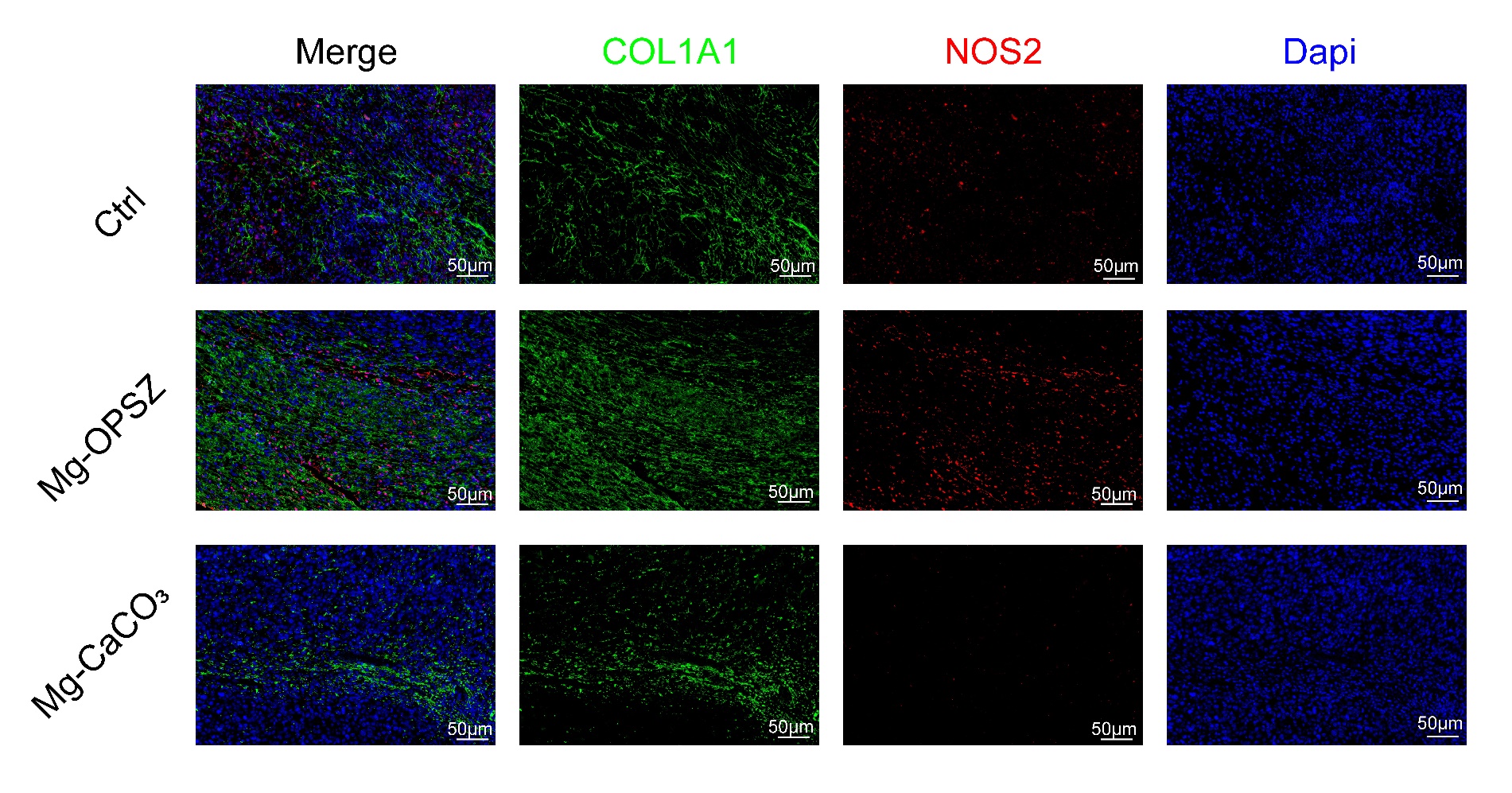


**Figure S19** IF images of tumors collected from Balb/c mice after different treatments. Green: COL1A1, red: NOS2, blue: DAPI, scale bar = 50 μm.


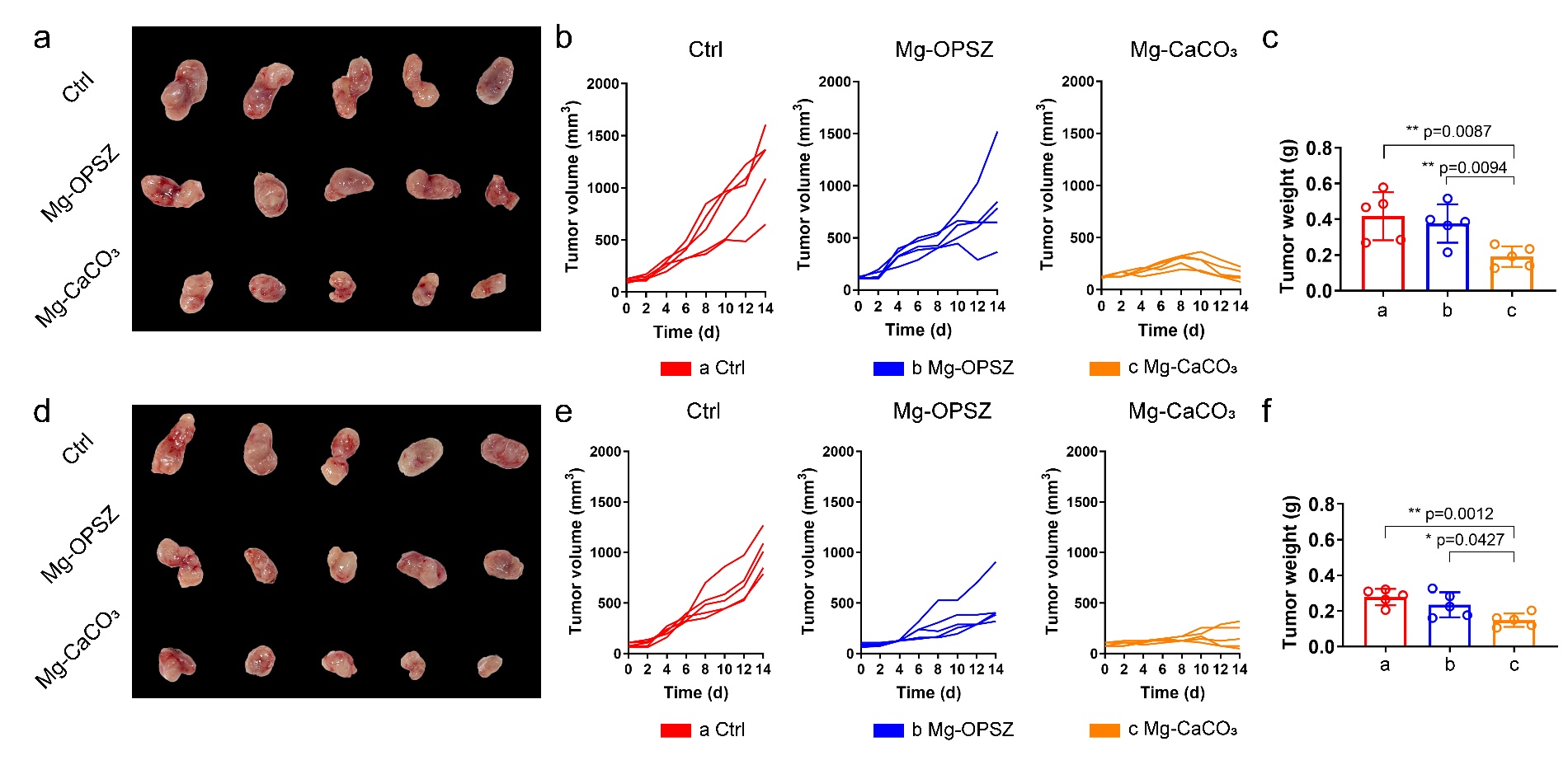


**Figure S20** (a, d) Representative photographs of primary tumors (a) and distant tumors (d) collected from Balb/c mice after different treatments. (b, e) Individual tumor growth curves of primary tumors (b) and distant tumors (e) of Balb/c mice after different treatments. (c, f) Tumor weight of primary tumors (c) and distant tumors (f) of Balb/c mice after different treatments (n = 5). Group: a: Ctrl, b: Mg-OPSZ, c: Mg-CaCO_3_. Statistical significance was calculated *via* two-tailed Students’ *t*-test. **p* < 0.05, ***p* < 0.01. The mean values and SD are presented.


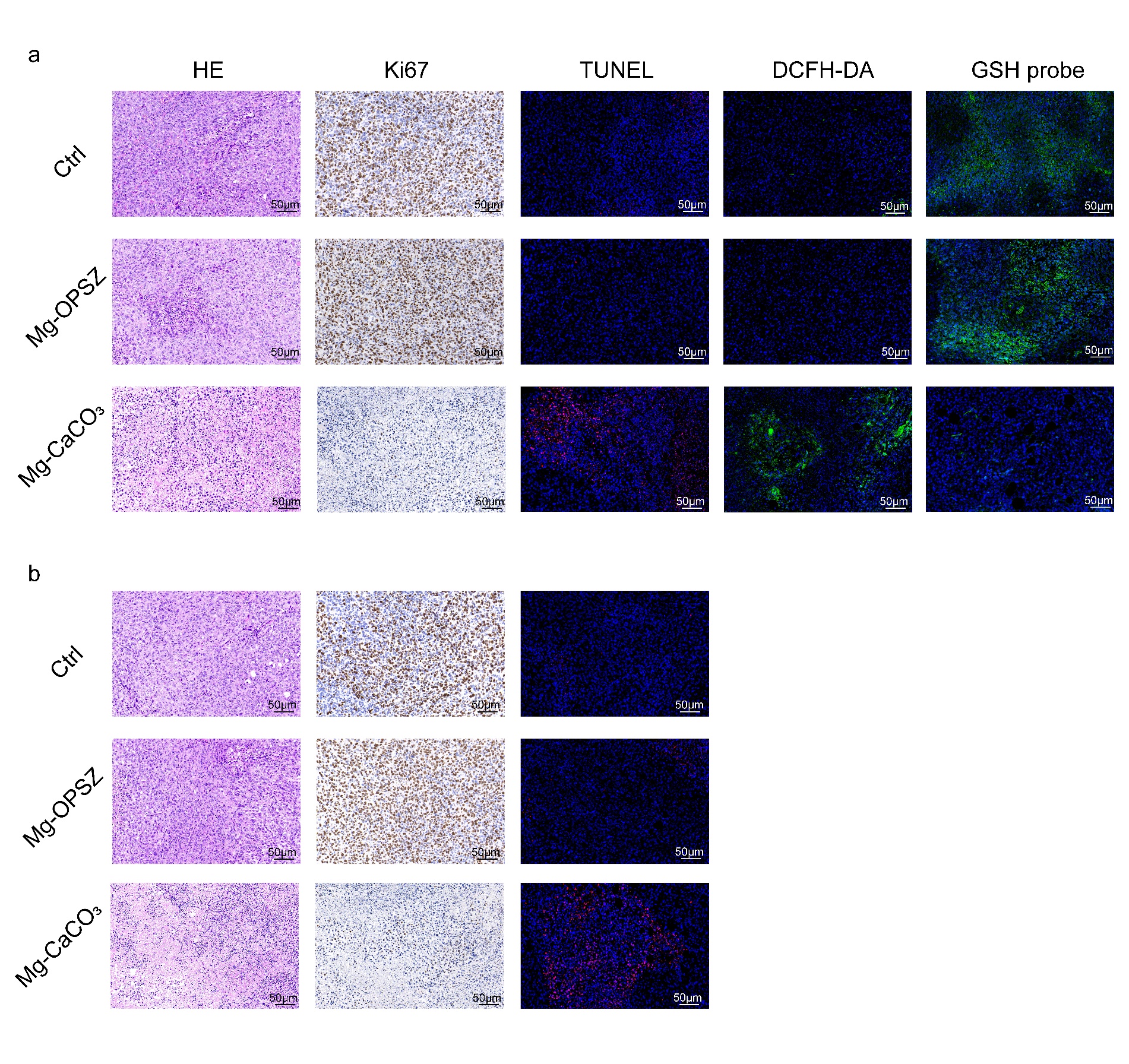


**Figure S21** (a) Microscopy images of H&E, Ki67, TUNEL, DCFH-DA, and GSH probe staining of primary tumors collected from Balb/c mice after different treatments. (b) Microscopy images of H&E, Ki67, and TUNEL staining of distant tumors collected from Balb/c mice after different treatments. Scale bar = 50 μm.


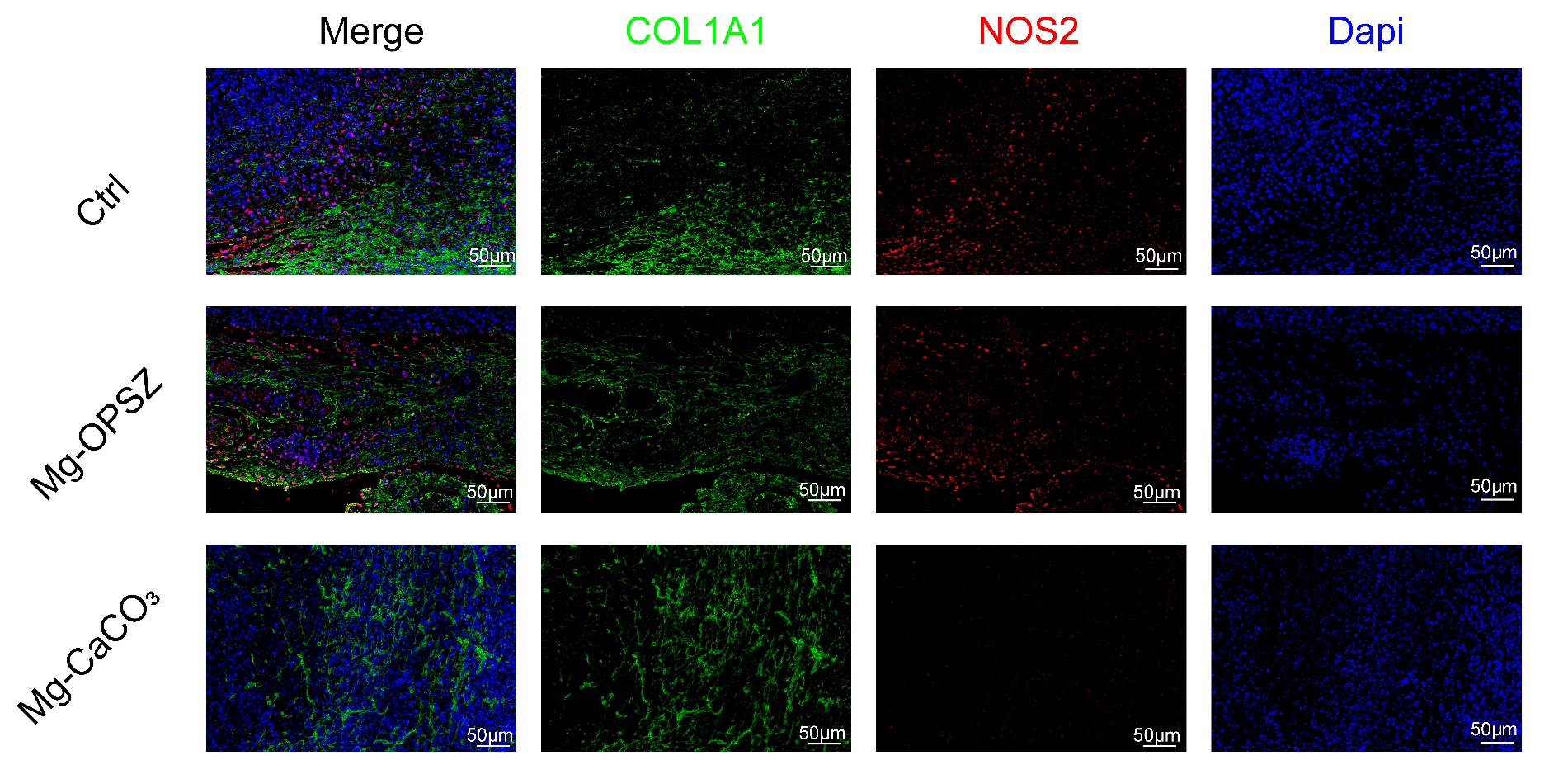


**Figure S22** IF images of primary tumors collected from Balb/c mice after different treatments. Green: COL1A1, red: NOS2, blue: DAPI, scale bar = 50 μm.


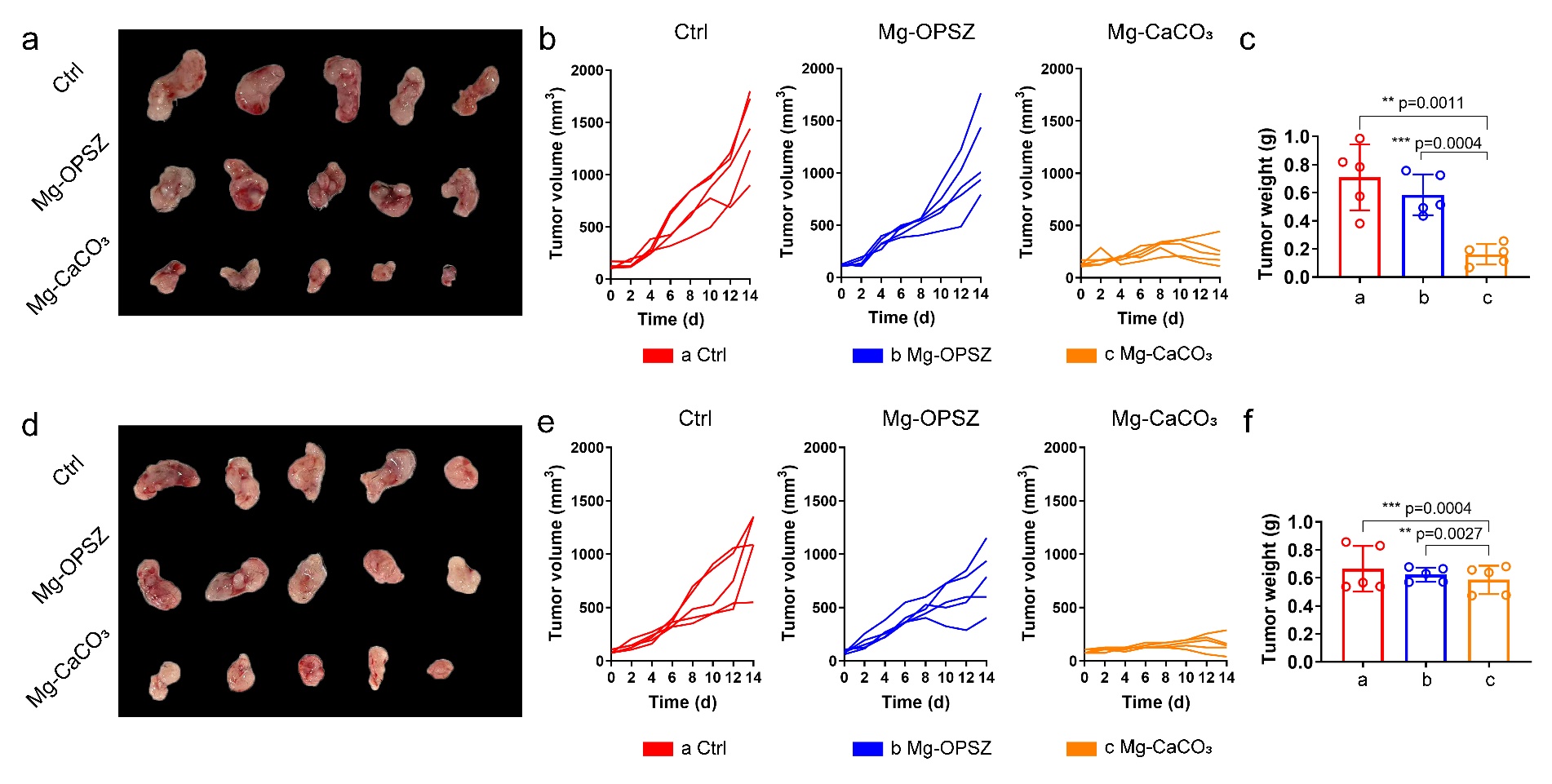


**Figure S23** (a, d) Representative photographs of primary tumors (a) and distant tumors (d) collected from C57BL/6 mice after different treatments. (b, e) Individual tumor growth curves of primary tumors (b) and distant tumors (e) of C57BL/6 mice after different treatments. (c, f) Tumor weight of primary tumors (c) and distant tumors (f) of C57BL/6 mice after different treatments (n = 5). Group: a: Ctrl, b: Mg-OPSZ, c: Mg-CaCO_3_. Statistical significance was calculated *via* two-tailed Students’ *t*-test. ***p* < 0.01, ****p* < 0.001. The mean values and SD are presented.


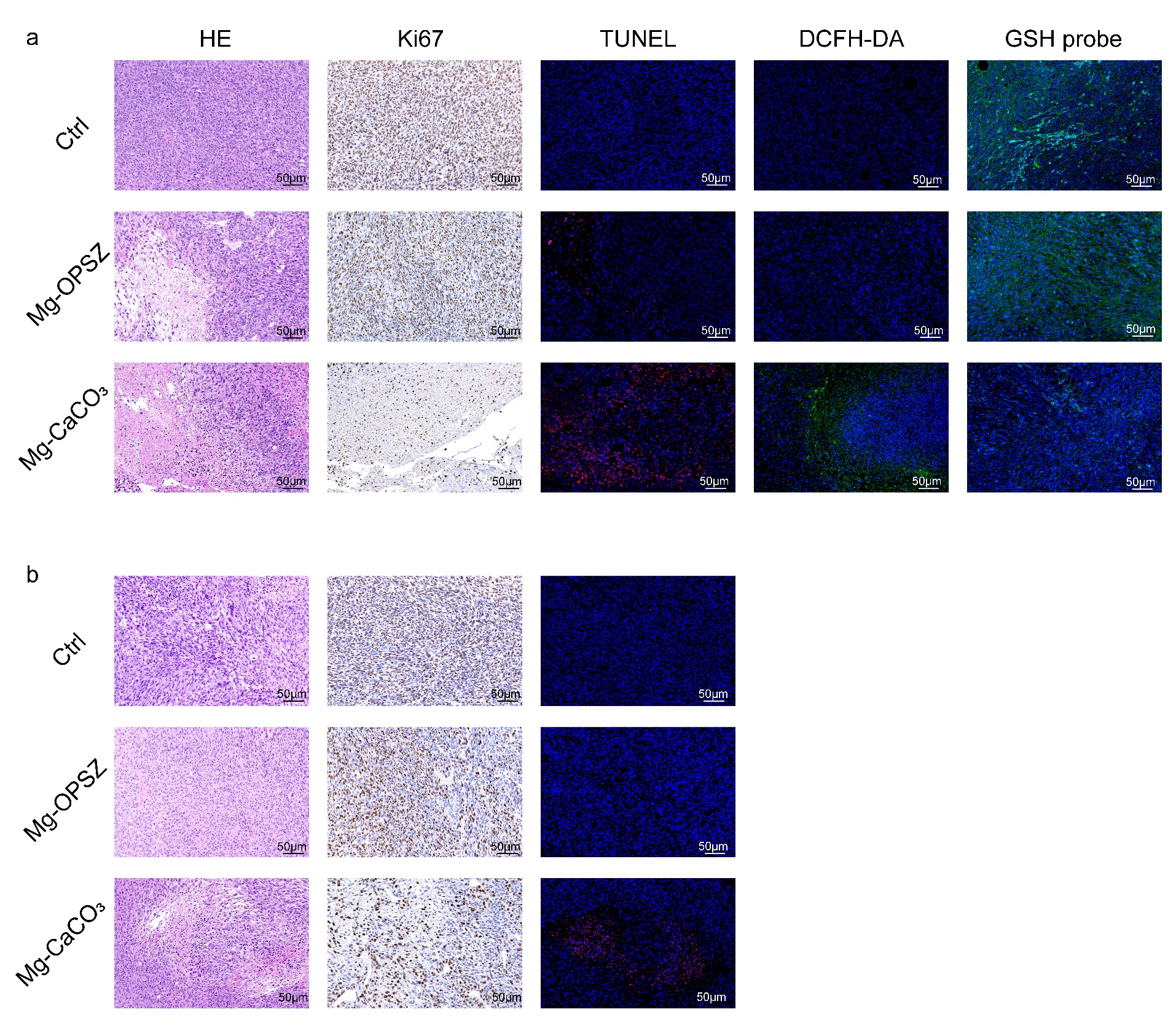


**Figure S24** (a) Microscopy images of H&E, Ki67, TUNEL, DCFH-DA, and GSH probe staining of primary tumors collected from C57BL/6 mice after different treatments. (b) Microscopy images of H&E, Ki67, and TUNEL staining of distant tumors collected from C57BL/6 mice after different treatments. Scale bar = 50 μm.


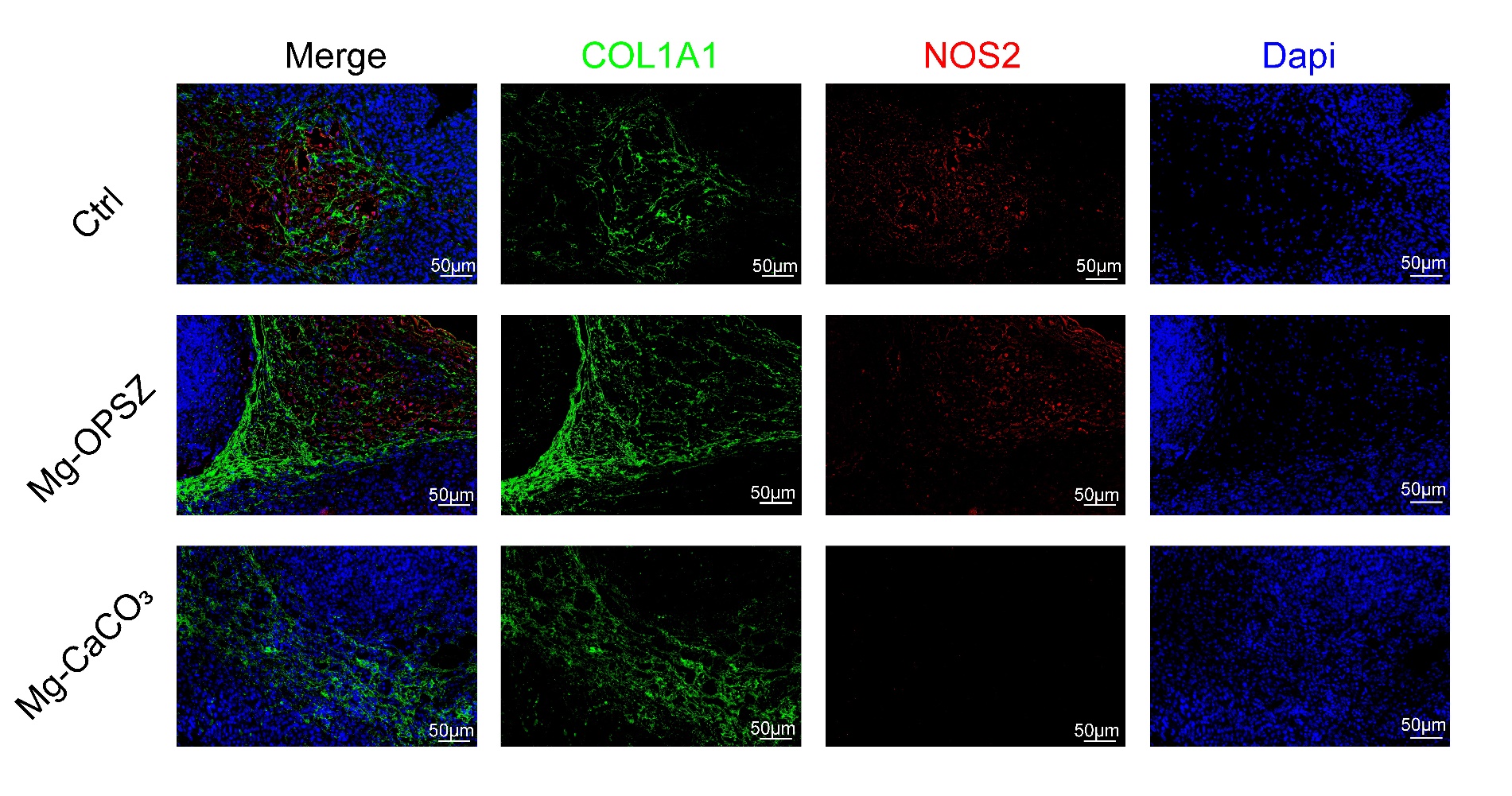


**Figure S25** IF images of primary tumors collected from C57BL/6 mice after different treatments. Green: COL1A1, red: NOS2, blue: DAPI, scale bar = 50 μm.


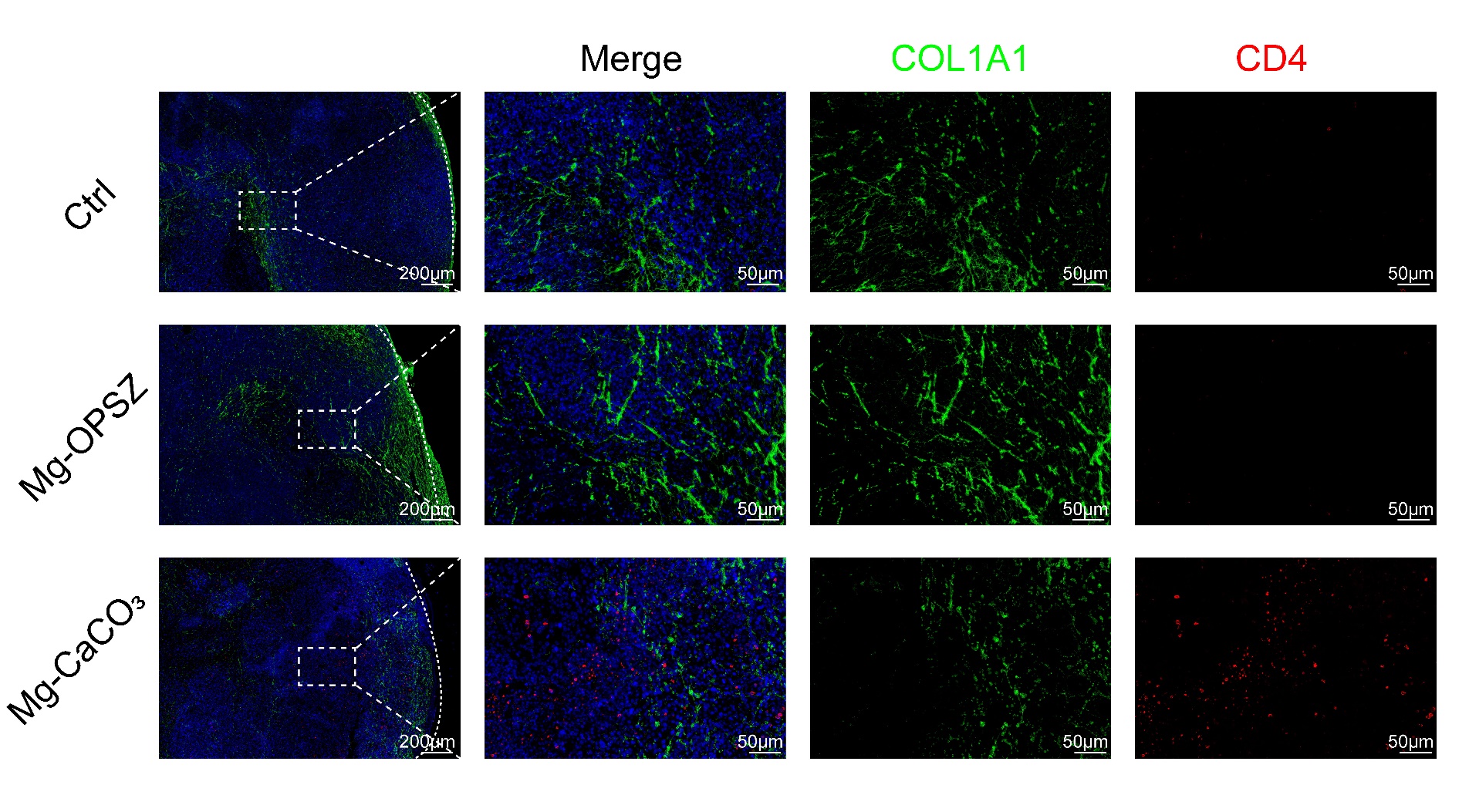


**Figure S26** IF images of primary tumors collected from Balb/c mice after different treatments. Green: COL1A1, red: CD4, blue: DAPI, scale bar: 200 μm and 50 μm, as indicated.


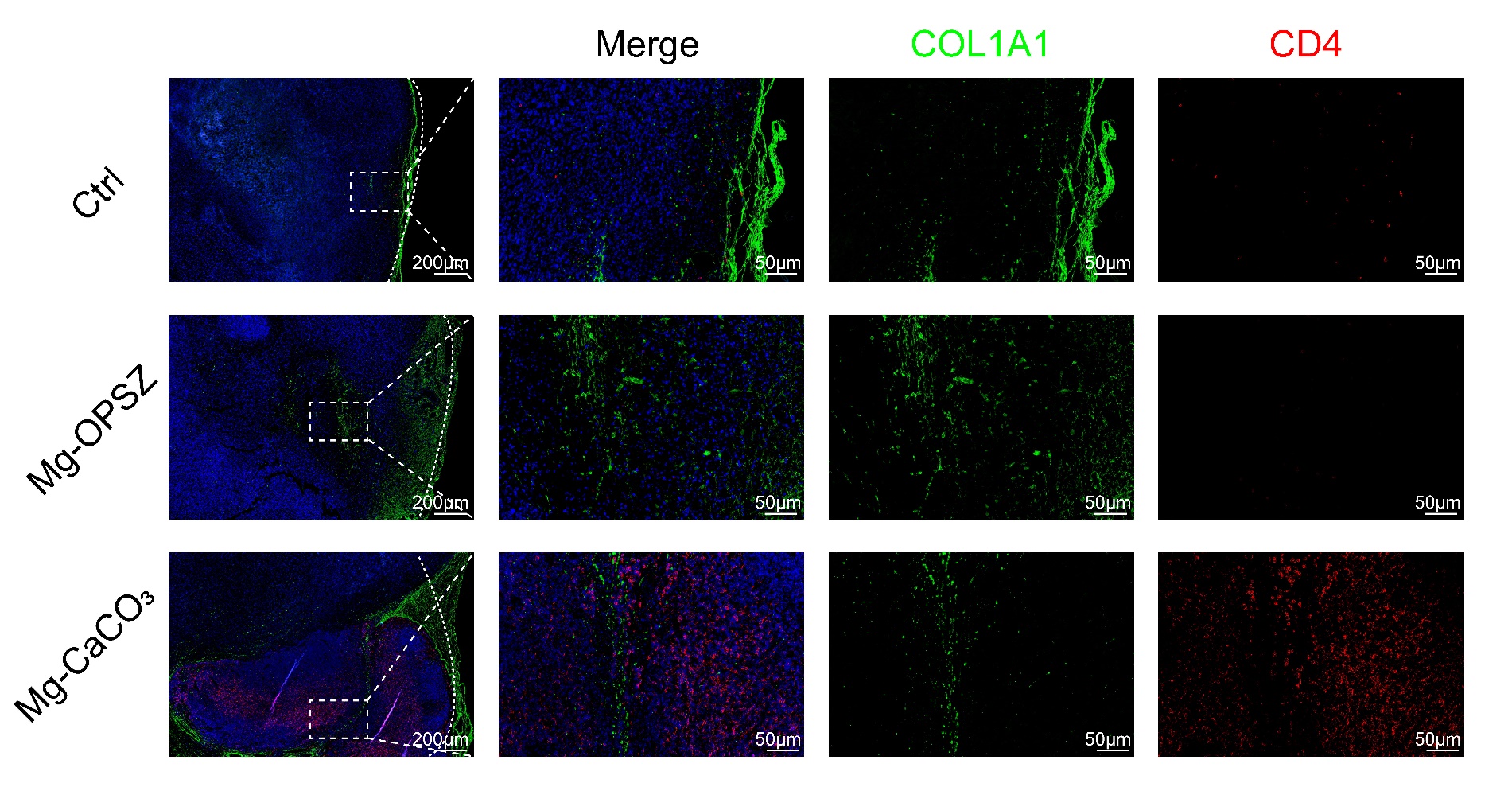


**Figure S27** IF images of primary tumors collected from C57BL/6 mice after different treatments. Green: COL1A1, red: CD4, blue: DAPI, scale bar: 200 μm and 50 μm, as indicated.


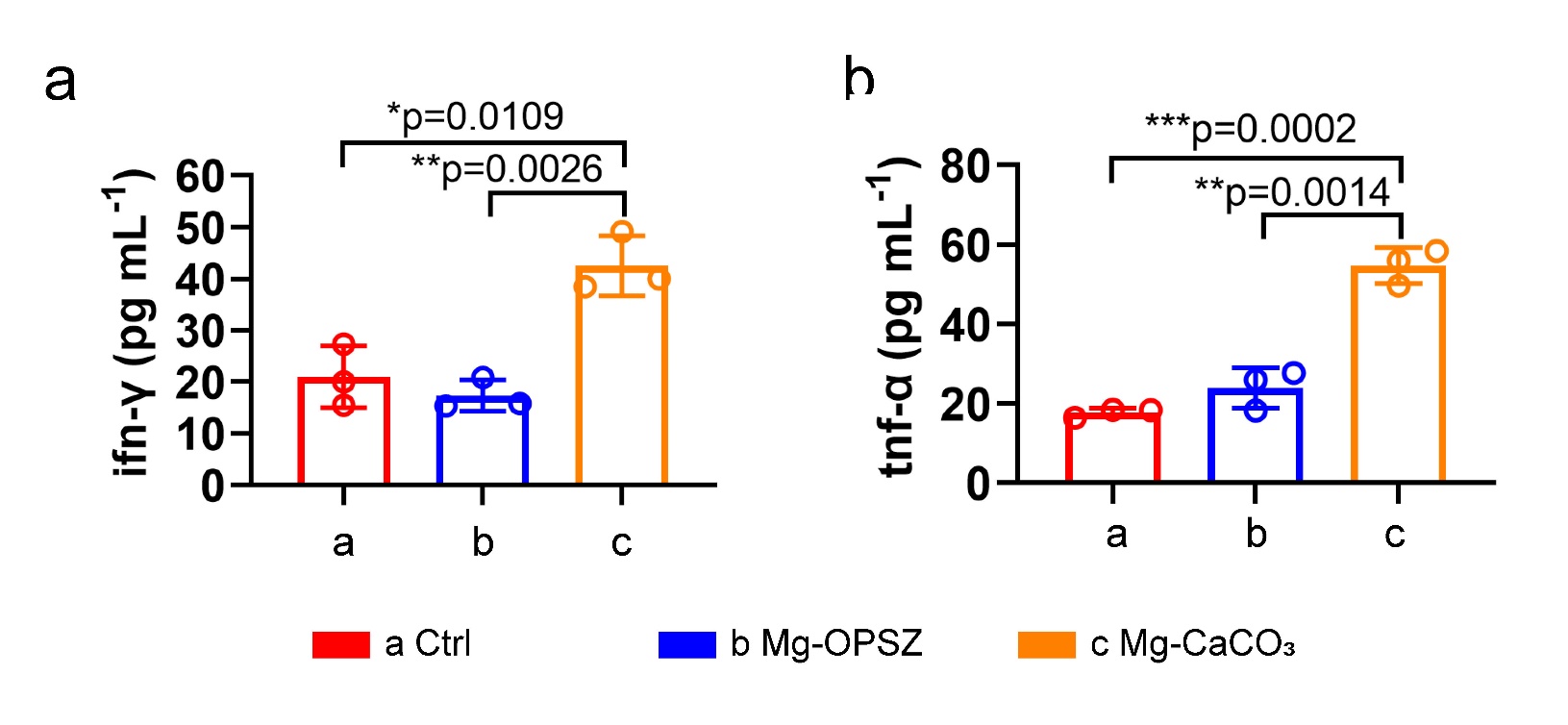


**Figure S28** Quantification of ifn-γ (a) and tnf-α (b) concentration in the serum of C57BL/6 mice after different treatments (n = 3). Group: a: Ctrl, b: Mg-OPSZ, c: Mg-CaCO_3_. Statistical significance was calculated *via* two-tailed Students’ *t*-test. **p* < 0.05, ***p* < 0.01 and ****p* < 0.001. The mean values and SD are presented.


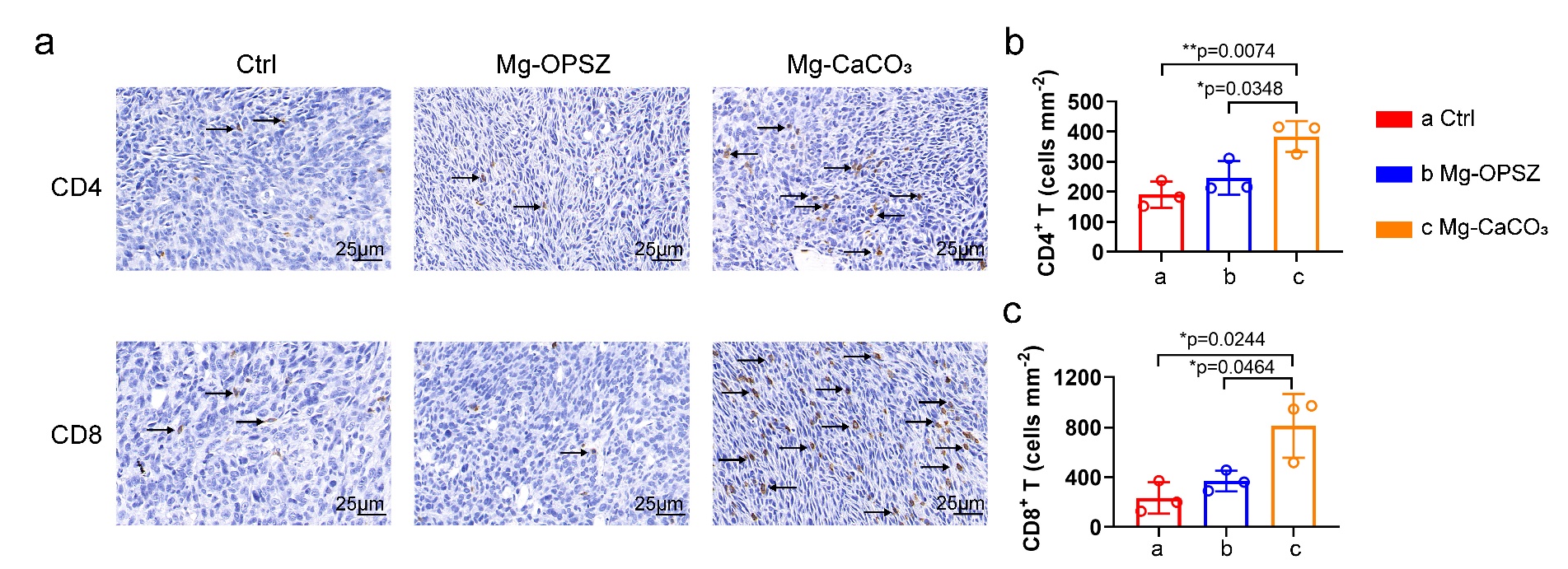


**Figure S29** (a) Representative IHC images of CD4^+^ and CD8^+^ T cell infiltration in distant tumors collected from C57BL/6 mice after different treatments. Scale bar = 25 μm. Quantification analysis of CD4^+^ (b) and CD8^+^ (c) T cell infiltration is showed in the right panel (n = 3). Group: a: Ctrl, b: Mg-OPSZ, c: Mg-CaCO_3_. Statistical significance was calculated *via* two-tailed Students’ *t*-test. **p* < 0.05 and ***p* < 0.01. The mean values and SD are presented.


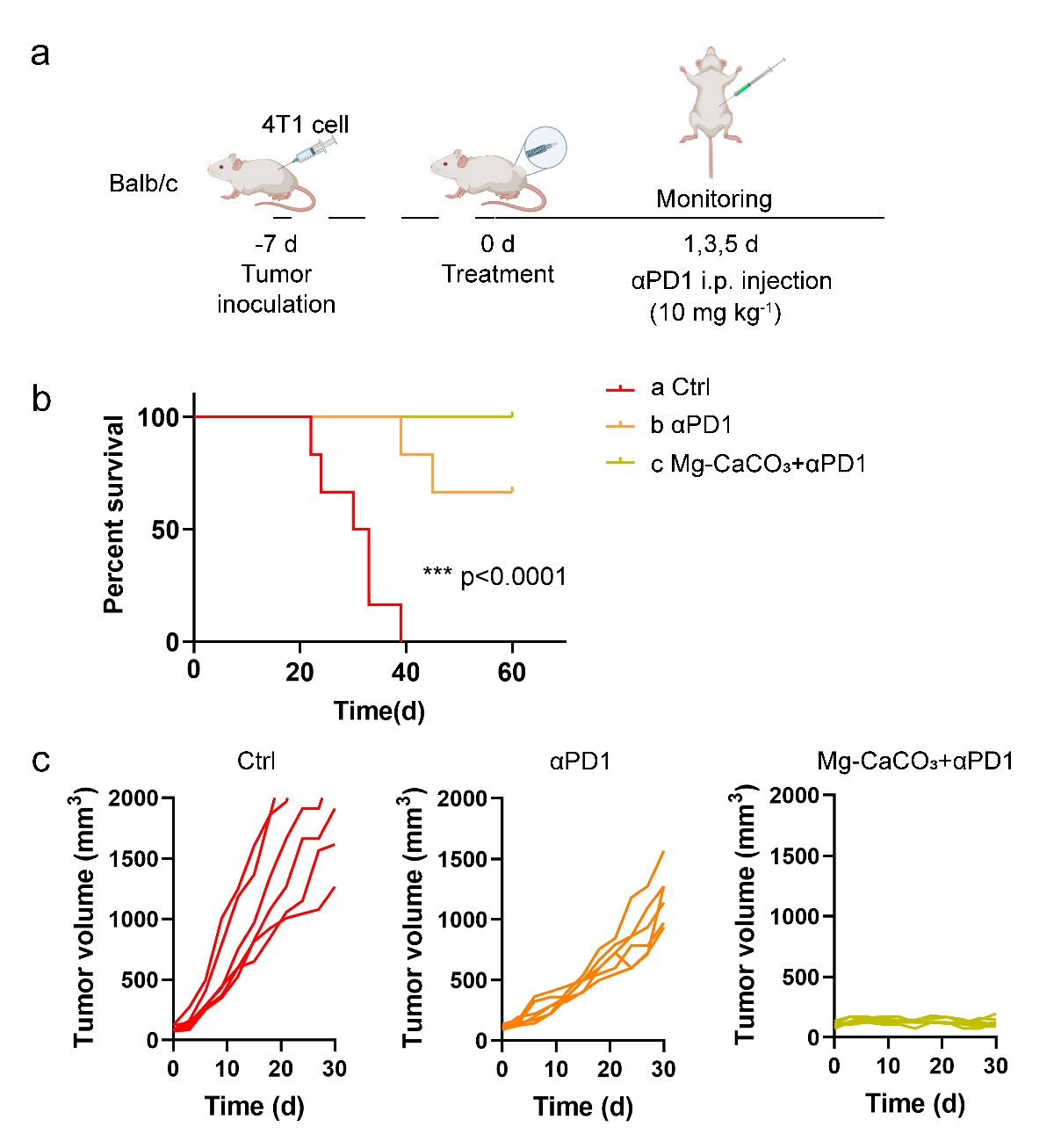


**Figure S30** (a) Schematic diagram of combination treatment (created with bioRender.com). (b) Survival curves of 4T1 tumor-bearing Balb/c mice (n = 6) after different treatment for 60 days. (c) Individual tumor growth curves of mice after various treatments. Group: a: Ctrl, b: αPD1, c: Mg-CaCO_3_+αPD1. Statistical significance was calculated *via* the log-rank test. ****p* < 0.001.

**Table S1**

| **Gene** | **Primer F'** | **Primer R'** |
| --- | --- | --- |
| Fn1 | TCTGTTGGGAGTGGTATCCTCTGTGC | TAAGTCTGGGTCACGGCTGTCTC |
| Col1a1 | GACAGGCGAACAAGGTGACAGAG | CAGGAGAACCAGGAGAACCAGGAG |
| Tgfβ1 | CCAGATCCTGTCCAAACTAAGG | CTCTTTAGCATAGTAGTCCGCT |
| Vim | CGTGCGGCTGCTTCAAGACTC | CTTCTCGTTGGTGCGGGTGTTC |
| Fap | GTATCTATGCTGGTCGCCTGTTGG | AGGTGGATCTCCTGGTCTTTGTTTC |
| Mmp2 | ACCATGCGGAAGCCAAGATGTG | AGGGTCCAGGTCAGGTGTGTAAC |
| Mmp11 | CGAGGTGGAGACTATTGGCGTTTC | TGGAAGGCAGCATCAATCTCAGAAG |
| Il6 | CTTCTTGGGACTGATGCTGGTGAC | TCTGTTGGGAGTGGTATCCTCTGTG |
| Cxcl1 | GGCTGGGATTCACCTCAAGAACATC | TGAGTGTGGCTATGACTTCGGTTTG |
| Cxcl5 | TGCGTTGTGTTTGCTTAACCGTAAC | TGACTTCCACCGTAGGGCACTG |
| Cxcl12 | AGAGCCACATCGCCAGAGC | ATCCACTTTAATTTCGGGTCAATGC |
| Lag3 | GCCATCTCGTTCTCGTTCTCATCC | TTCTCCACCAGTGAAAGCCAAAGG |
| Foxp3 | AATGCCATCCGCCACAACCTG | ACACTGCTCCCTTCTCGCTCTC |
| Nkg7 | CCAACAAGCCAAGAGACTCAAGTAG | ACAGGACAGCCAGGATACAGAAG |
